# Effects of post-stress corticosterone on hippocampal excitability and behavior involving hyperpolarization-activated cation channel 1 function

**DOI:** 10.1038/s41398-026-03871-4

**Published:** 2026-02-07

**Authors:** Chung Sub Kim, Jiwon Kim, Sandali Michael

**Affiliations:** Department of Neuroscience & Regenerative Medicine, Medical College of Georgia at Augusta University, Augusta, GA 30912 USA

**Keywords:** Hippocampus, Molecular neuroscience

## Abstract

Single Prolonged Stress (SPS) is a widely used rodent model for investigating the consequences of acute traumatic stress, but outcomes in mice are often variable across strains and behavioral domains. Because corticosterone (CORT) release is a central feature of the stress response, we combined SPS with post-stress CORT administration (SPS + CORT) to capture this hormonal component and unmask latent phenotypes. Hyperpolarization-activated cyclic nucleotide-gated 1 (HCN1) channels are highly expressed in the dorsal CA1 (dCA1) hippocampus, where they regulate neuronal excitability. We previously demonstrated that acute CORT enhances hyperpolarization-activated current (*I*_h_) in vitro; here, we tested its in vivo contribution to stress-related behavioral and physiological outcomes. Male mice (8–9 weeks old) were exposed to SPS followed by vehicle or CORT. Behavioral assays—including the open field, Y-maze, and contextual fear conditioning—revealed that SPS + CORT mice displayed impaired spatial working memory and deficits in contextual recall and fear extinction, resembling core PTSD-like features. Whole-cell recordings from dCA1 neurons showed decreased input resistance, reduced action potential firing, and elevated *I*_h_, which were normalized by the HCN channel blocker ZD7288. Overexpression of HCN1 in SPS mice reproduced both behavioral and physiological phenotypes seen in SPS + CORT mice, whereas genetic deletion of HCN1 in SPS + CORT mice reduced *I*_h_ and rescued the behavioral abnormalities. Together, these findings identify HCN1 channels as a critical mediator linking post-stress glucocorticoid signaling to maladaptive hippocampal plasticity and PTSD-like outcomes.

## Introduction

After being exposed to traumatic events, people can develop post-traumatic stress disorder (PTSD), a crippling mental illness marked by heightened arousal, avoidance, negative changes in mood and cognition, and persistent re-experiencing [1]. There is evidence of hippocampal atrophy and impaired memory processing in those who have PTSD, and an increasing amount of neuroimaging and clinical research has linked dysfunction in the hippocampus, amygdala, and prefrontal cortex as key neural substrates of the disorder [2–4]. To model these features experimentally, several rodent paradigms have been established. Among them, the single prolonged stress (SPS) protocol - combining restraint, forced swim, and anesthetic exposure - has been widely used, especially in rats [5, 6], where it produces reliable changes reminiscent of PTSD, such as enhanced anxiety, impaired extinction of fear memories, and dysregulation of the hypothalamic–pituitary–adrenal (HPA) axis [5–7]. In mice, however, outcomes have been less consistent. Results vary depending on the genetic background, the behavioral domains tested, and whether modifications were made to the original procedure [8–10]. For example, a multimodal SPS protocol that included predator odor failed to alter open field or elevated plus maze behavior, though other cognitive domains were not assessed [8]. Another study using a “stress–restress” approach reported anxiety-like effects in C57BL/6 mice but not in wild-derived strains, again without evaluating memory outcomes [9]. In contrast, a recent report showed that canonical SPS can produce robust behavioral effects when combined with repeated baseline testing, suggesting that additional stressors may sensitize mice to the paradigm [10]. Taken together, these findings indicate that while SPS reliably induces PTSD-like phenotypes in rats, in mice it often generates a latent vulnerability that is not consistently expressed unless paired with further challenges. Stress hormones are one such factor that could reveal this vulnerability. Glucocorticoids, particularly corticosterone (CORT) in rodents, are essential components of the stress response and exert profound effects on hippocampal circuits [11, 12]. Elevated CORT has been shown to disrupt contextual memory and, when infused into the dorsal CA1 region, produces contextual amnesia alongside exaggerated recall of trauma-related cues [13, 14]. Our earlier work demonstrated that acute CORT exposure increases the expression of hyperpolarization-activated cyclic nucleotide-gated channel 1 (HCN1) in dorsal CA1 pyramidal neurons and enhances the corresponding hyperpolarization-activated current (*I*_h_) through a glucocorticoid receptor–HCN–PKA signaling pathway [15]. Importantly, HCN1 channels have been linked to a range of stress-related outcomes in other rodent models, including chronic unpredictable stress and chronic social defeat stress [16–19], where they influence neuronal excitability, key molecular markers including brain-derived neurotrophic factor and mammalian target of rapamycin, synaptic plasticity, and behavioral responses [15, 17, 18, 20, 21]. Building on this background, we sought to determine whether supplementing canonical SPS with post-stress CORT administration could amplify glucocorticoid signaling and uncover mechanistic links between stress hormones and hippocampal dysfunction. Using this approach, we found that SPS combined with CORT produced consistent deficits in spatial working memory and contextual fear extinction, along with HCN1-dependent alterations in dorsal CA1 excitability. These impairments were prevented by genetic deletion of HCN1 and mimicked by HCN1 overexpression, identifying HCN1 as a key mediator of maladaptive hippocampal plasticity following stress hormone exposure.

## Materials and methods

### Animals

Male C57BL/6 J and HCN1^flox/flox^ mice aged 7 to 10 weeks were used in this study. Mice were housed 2-5 per cage under a 12-hr light/dark cycle (lights on at 6 am, off at 6 pm) with *ad libitum* access to water and food. All procedures involving animals were approved by the Institutional Animal Care and Use Committee of Augusta University.

### Drugs and viruses

Catalog numbers and manufacturers are listed in the Supplementary Information.

### Single prolonged stress procedures

The single prolonged stress (SPS) protocol consists of three sequential stressors: 2-hr restraint, 15-min forced swim, and exposure to isoflurane anesthesia (2%). Briefly, mice were restrained for 2 hr in a small acrylic tube (50 ml). Immediately afterward, the mice underwent a 15-min forced swim stress in a transparent Plexiglass cylinder (25 cm tall x 20 cm in diameter) filled with water (23 °C - 24 °C) to a depth of 15 cm. Following the swim, mice were dried by a towel and exposed to 2% isoflurane for 5 min using a soaked cotton pad in a closed plastic container. After completion of SPS, mice were singly housed and left undisturbed for 10 days. This undisturbed single-housing period is a standard feature of the SPS paradigm, as it allows for the consolidation and incubation of stress responses, which is critical for the later emergence of PTSD-like phenotypes [6, 7, 22].

### Behavioral experiment

Social defeat stress and related behavioral assays were conducted between 4 and 6 pm to align with the animals’ active phase. Contextual fear conditioning was performed between 10 am and 2 pm, a period when circulating corticosterone levels are relatively low, to minimize variability in stress hormone responses [23]. These testing windows are consistent with our previous work [15, 18, 24], ensuring methodological continuity across studies.

### Open field test

Anxiety-like behavior and locomotor activity were assessed using the open field test as described previously [18]. Each mouse was placed in the center of the open field arena and allowed to explore freely for 6 min while being recorded by a CCD camera. The arena was cleaned with 70% ethanol between trials to eliminate odor cues. Key parameters measured included total distance traveled, number of entries into the center zone (50% of the total surface area), and the duration spent in the center. A line crossing was counted when the mouse’s body center crossing a demarcation line.

### Y-maze spontaneous alternation test

Short-term spatial working memory was evaluated with the Y-maze spontaneous alternation test, as described previously [15, 18]. The maze was constructed from opaque white Plexiglas and consisted of three identical arms (14 in × 3 in, wall height 12 cm) arranged at 120° angles, with no intra-maze cues or arm blocking. The apparatus was placed in a dimly red-lit room (3 – 4 lux at the maze center), and extramaze visual cues were minimized. Mice were placed at the end of one arm and allowed to explore freely for 6 min. An arm entry was defined by all four paws entering an arm, and sequences were recorded using an overhead CCD camera. Alternation percentage was calculated as the ratio of actual alternations to the maximum possible (total entries minus two). Additional metrics included total arm entries and self-arm return (SAR), defined as re-entering the same arm after exiting. SAR was quantified to assess perseverative behavior, as our previous studies have shown that HCN1 upregulation and increased *I*_h_ are associated with reduced spontaneous alternations and increased SAR in socially defeated mice [15, 18]. The maze was cleaned with 70% ethanol between animals.

### Contextual fear conditioning, fear memory, and fear extinction

To assess fear memory and extinction, a tone-shock unpairing protocol was used as described previously [13, 18]. On Day 1 (habituation), mice were exposed for 4 min to a neutral chamber (context B) with an opaque PVC floor. On Day 2 (conditioning), animals were placed in a distinct chamber (context A; 24 × 24 cm) equipped with stainless steel rod floors for shock delivery. During the 4 min session, two 1 kHz tones (65 dB, 15 s) and two foot shocks (0.6 mA, 1 s) were presented in a pseudo-random order, separated by variable intervals (20–30 s), such that the tone and shock were not temporally paired. This unpairing procedure emphasizes the contextual predictors of shock rather than the tone. 24 h later (Day 3), memory retrieval was assessed through two separate tests. First, mice were returned to context B for a tone re-exposure test lasting 6 min. This session was divided into three 2-minute phases: pre-tone, tone presentation, and post-tone. Freezing behavior, defined as the absence of all movement except respiration, was quantified during each phase using FreezeView 5 software (Actimetrics, USA). The freezing data were then used to calculate a tone ratio reflecting conditioned fear responses while controlling for baseline activity. 2 h after the tone test, mice underwent a context re-exposure test in context A for 6 min, where freezing behavior was again monitored to assess memory of the conditioning environment. Fear extinction was evaluated in subsequent sessions where mice were placed in context A for 6 min daily across three consecutive days. Freezing behavior during these sessions was tracked to measure the reduction of conditioned fear responses over time. Throughout all behavioral testing phases, continuous white noise at 55 to 60 dB was maintained to mask any extraneous environmental sounds, thereby minimizing confounding auditory stimuli and helping to ensure that behavioral responses were specific to the experimental manipulations. The tone ratio is calculated as follows: [% freezing during tone presentation − (% pre-tone period freezing + % post-tone period freezing)/2]/[% freezing during tone presentation + (% pre-tone period freezing + % post-tone period freezing)/2] [13, 18]. The 1 kHz, 65 dB tone was selected based on prior studies [13, 18], where it was used as a neutral auditory cue. Consistent with these reports, under our conditions the tone did not induce freezing in control animals, although mice are fully capable of detecting this frequency [25]. All experiments were conducted with investigators blinded to treatment groups.

### Stereotaxic microinjection

Stereotaxic microinjections were carried out following established protocols with minor modifications [18, 26], with detailed methodology provided in the Supplementary Information.

### Acute hippocampal slice preparation

Mice were anesthetized with isoflurane gas (2-3%) in a closed plastic container and transcardially perfused with ice-cold artificial cerebral spinal fluid (aCSF) composed of (in mM): 2.5 KCl, 1.25 NaH_2_PO_4_, 25 NaHCO_3_, 0.5 CaCl_2_, 7 MgCl_2_, 7 dextrose, 210 sucrose, 1.3 ascorbic acid, and 3 sodium pyruvate, bubbled with 95% O_2_ - 5% CO_2_. The brain was removed and hemisected along the longitudinal fissure. Dorsal hippocampal slices were prepared as previously described [15, 18]. 300 μm thick hippocampal slices were made in ice-cold aCSF using a vibrating microtome (Microslicer DTK-Zero1, DSK, Kyoto, Japan). Slices were placed in a holding chamber containing (in mM) 125 NaCl, 2.5 KCl, 1.25 NaH_2_PO_4_, 25 NaHCO_3_, 2 CaCl_2_, 2 MgCl_2_, 12.5 dextrose, 1.3 ascorbic acid, and 3 sodium pyruvate, bubbled with 95% O_2_ - 5% CO_2_ at 35 °C for 30 min and then incubated for at least 45 min at room temperature before used for electrophysiology. Whole-cell patch-clamp recordings were performed as previously described [15, 18]. Briefly, hippocampal slices were submerged in a recording chamber continuously perfused with aCSF containing (in mM) 125 NaCl, 3 KCl, 1.25 NaH_2_PO_4_, 25 NaHCO_3_, 2 CaCl_2_, 1 MgCl_2_, and 12.5 dextrose, bubbled with 95% O_2_ - 5% CO_2_ at a rate of 1 ml/min and 31-33°C. CA1 pyramidal neurons were visually identified using a microscope (Olympus BX51WI, US) fitted with differential interference contrast optics [27]. Patch pipettes for somatic (4–7 MΩ) were prepared with capillary glass (external diameter 1.65 mm and internal diameter 1.1 mm, World Precision Instruments) using a Flaming/Brown micropipette puller (P-1000, Sutter Instrument, CA) and filled with an internal solution containing (in mM) 120 K-gluconate, 20 KCl, 10 HEPES, 4 NaCl, 7 K2-phosphocreatine, 4 Mg-ATP, 0.3 Na-GTP (pH 7.3 with KOH). Whole-cell patch-clamp recordings were conducted using a MultiClamp 700B amplifier (Molecular Devices, LLC., CA) and acquired with pCLAMP10 software (Molecular Devices, LLC., CA). Electrical signals were filtered at 10 kHz, sampled at 20 kHz, digitized by Axon Digidata 1440 A (Axon Instruments), and analyzed offline. For whole-cell current-clamp recordings, series resistance was continuously monitored; recordings were excluded if the series resistance exceeded 15 MΩ during the recordings. All recordings were performed without synaptic blockers, as dorsal CA1 pyramidal neurons under our conditions did not exhibit spontaneous synaptic activity or rhythmic firing. Resting membrane potential was defined as the membrane potential recorded in the absence of injected current. Liquid junction potential was not corrected but was estimated to be approximately -13 mV using the Patcher’s Power Tools plugin in Igor Pro.

### Immunohistochemistry

Immunohistochemistry was carried out as described previously [15, 18], with detailed methodology provided in the Supplementary Information.

### Data Analysis

Input resistance was measured by the slope of the linear fit of the V-I plot between +30 and -150 pA current injections. Electrophysiological data were analyzed in Easy Electrophysiology and Axograph.

### Statistical analysis

Statistical analyses were performed using GraphPad Prism (version 10). The specific test applied to each dataset is indicated in the figure legends. For experiments with a single independent variable (e.g., SPS-Veh, SPS-CORT, SPS-HCN1), data were analyzed using one-way ANOVA with Tukey’s or Dunnett’s post hoc tests, or by Kruskal–Wallis with Dunn’s correction when data violated normality assumptions (Shapiro–Wilk, D’Agostino–Pearson, Anderson–Darling, Kolmogorov–Smirnov). For experiments with two independent variables (e.g., stress × virus, stress × treatment), two-way ANOVA was used to assess main effects and interactions, followed by Tukey’s or Sidak’s multiple comparisons tests. Paired or unpaired t-tests (or nonparametric equivalents, Wilcoxon signed-rank or Mann–Whitney U tests) were applied where appropriate. All data are presented as mean ± SEM. For electrophysiological recordings, individual cells were treated as the experimental unit. The total number of cells and the number of animals from which they were derived are reported in Supplementary Information (Table S1). The average number of cells per animal did not differ significantly between groups. Effect sizes and test statistics (F, t, U, or W values, with degrees of freedom) are reported in the Supplementary Information. Sample sizes were determined based on prior studies using similar behavioral and electrophysiological paradigms [15, 18, 22, 24], which established variance ranges and effect sizes sufficient to detect biologically meaningful differences. Group sizes for both behavioral and electrophysiological experiments are detailed in the Supplementary Information (Table S1).

### Ethics approval and consent to participate

All experimental procedures were performed in accordance with the relevant guidelines and regulations. All animal experiments were approved by the Institutional Animal Care and Use Committee (IACUC) of Augusta University (approval number: [IACUC-2020-1031) and were conducted in compliance with the National Institutes of Health Guide for the Care and Use of Laboratory Animals.

## Results

### SPS-CORT mice display impaired short-term working memory, contextual amnesia, and impaired fear extinction

**Fig. 1:**
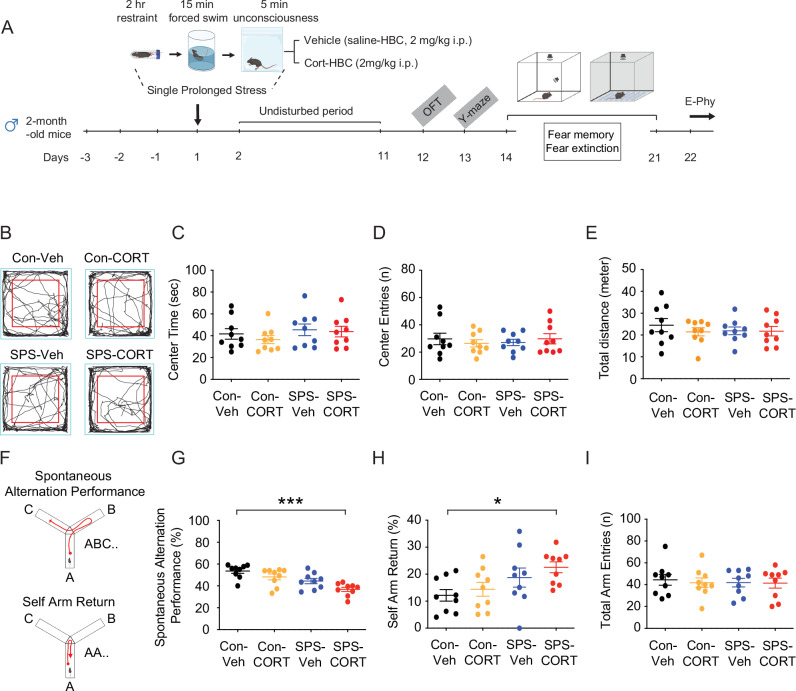
SPS-CORT mice exhibit impaired short-term spatial working memory. (**A**) Schematic diagram of the experimental design for single prolonged stress, behavioral tests, and electrophysiology. (**B**) Representative video tracking images of age-matched male mice during open field test. (**C**) Center time. (**D**) Center entries. (**E**) Total traveled distance. (**F**) Illustration of the spontaneous alternation Y maze test. (**G**) Percentage of spontaneous alternation performance among groups. (**H**) Percentage of self-arm return. (**I**) Total arm entries. The Kruskal-Wallis test was performed followed by Dunn’s multiple comparisons test in (**G**) and (**H**). Data are expressed as mean ± SEM (animal n values provided in Supplementary Table 1). *P < 0.05 and ***P < 0.001. Further statistical information is provided in the Supplementary Table 2. Panel A was created with BioRender.com.

**Fig. 2:**
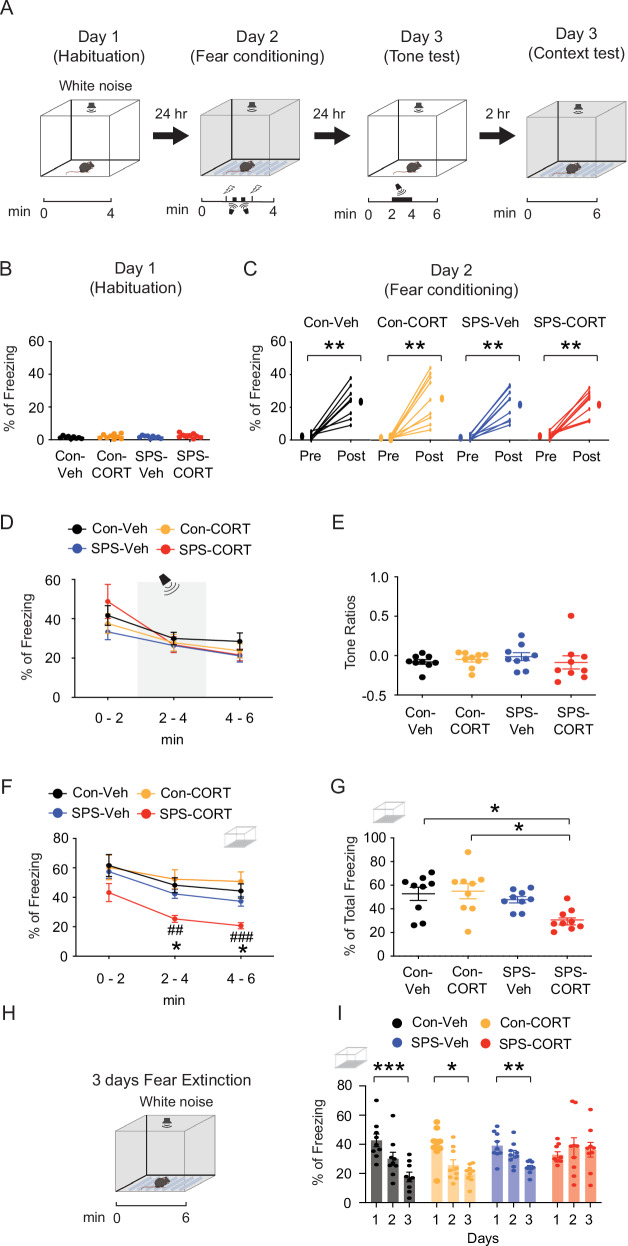
SPS-CORT mice exhibit contextual amnesia and impaired fear extinction. (**A**) Illustration showing the contextual fear conditioning with the CS-US unpairing protocol in control or SPS mice with Veh or CORT treatment. (**B**) Percentage of freezing during the habituation phase. (**C**) Fear acquisition on Day 2 was measured both before (first 1 min) and after (last 1 min) the session. The Wilcoxon matched-pairs signed rank test was performed separately for the control-Veh, control-CORT, SPS-Veh, and SPS-CORT groups. (**D**) Percentage of freezing during the tone memory test. The tone was present during the 2-4-minute session of the experiment. (**E**) Tone ratios. (**F**) Percentage of freezing during the context memory test. The Two-Way ANOVA followed by Tukey’s post-hoc test was performed. * indicates a comparison between the control-Veh and the SPS-CORT group. ^#^ indicates a comparison between the control-CORT and the SPS-CORT group. (**G**) The total percentage of freezing during a 6-minute context memory test. (**H**) Illustration of fear extinction experiment. (**I**) Fear extinction during the 3-day fear extinction test. The mouse was placed into the context for 6 min per day for 3 consecutive days. The Kruskal-Wallis test was performed followed by Dunn’s multiple comparisons test separately for the control-Veh, control-CORT, SPS-Veh, and SPS-CORT groups. Data are expressed as mean ± SEM (animal n values provided in Supplementary Table 1). *P < 0.05, **P < 0.01, ***P < 0.001, ^##^*P* < 0.01, and ^###^*P* < 0.001. Further statistical information is provided in the Supplementary Table 2. Panel A and H were created with BioRender.com.

Two-month-old male mice were subjected to the SPS procedure, while age-matched controls remained unexposed (Fig. 1A). Given that acute corticosterone treatment has been shown to upregulate HCN1 protein expression and enhance *I*_h_ in vitro [15], we asked whether post-SPS corticosterone administration would influence behavioral outcomes. Mice received either vehicle (saline-HBC; 2 mg/kg, intraperitoneal [i.p.]) or corticosterone (CORT-HBC; 2 mg/kg, i.p.) immediately following the SPS procedure (Fig. 1A). In rodent models, particularly in rats, a recovery period of at least 7 days is considered critical for the emergence of PTSD-like phenotypes, including heightened anxiety, depressive-like behavior, hyperarousal, social withdrawal, impaired fear extinction and cognition, and HPA axis dysregulation [28]. Following a 10-day undisturbed period, we performed a battery of behavioral assessments. In the open field test (Fig. 1B), no significant group differences were observed in center time (Fig. 1C), center entries (Fig. 1D), or total distance traveled (Fig. 1E), indicating no gross locomotor or anxiety-like alterations. However, in the Y-maze test (Fig. 1F), SPS-CORT mice exhibited significantly reduced spontaneous alternation performance (SAP; Fig. 1G) and increased same-arm returns (SAR; Fig. 1H), while the total number of arm entries remained unchanged (Fig. 1I), suggesting deficits in spatial working memory rather than changes in exploratory activity. We next assessed fear memory and extinction using a contextual fear conditioning paradigm with an unpaired presentation of the conditioned stimulus (CS) and unconditioned stimulus (US) (Fig. 2A). Our use of an unpaired CS–US protocol was an intentional design choice to isolate hippocampus-dependent contextual fear learning, in line with prior PTSD models [13, 14, 18]. To validate this approach.

We directly compared control mice undergoing paired versus unpaired CS–US procedures with identical tone and shock parameters (Figure S1A). As expected, the paired group showed stronger freezing than the unpaired group; however, the unpaired group still exhibited a significant increase in freezing from pre- to post-conditioning (Figure S1C), demonstrating reliable contextual fear learning in the absence of tone-shock contingency. Moreover, unpaired mice displayed robust contextual freezing during recall (Figures S1H and S1I) and progressive reductions in freezing across extinction sessions (Figure S1K), confirming both acquisition and extinction of contextual fear memory. In this protocol, the context served as the CS and the foot shock as the US, while the tone was presented in an unpaired manner and did not predict the shock. As a result, the context became the primary predictor of the aversive event, driving the freezing response [13, 18]. Having established the validity of this paradigm, we examined fear memory and extinction across groups. No significant differences were observed among the groups during habituation on Day 1 (Fig. 2B) or fear acquisition on Day 2 (Fig. 2C). On Day 3, mice underwent a tone re-exposure test to evaluate tone-associated freezing responses. As anticipated under the CS–US unpairing protocol, the tone did not serve as a predictive cue, resulting in no significant increase in freezing during tone presentation (Fig. 2D) Consistently, there were no group differences in freezing behavior during the tone test (Fig. 2D) or in tone discrimination ratios (Fig. 2E). Two hours later, mice were re-exposed to the original conditioning context in which they had previously received foot shocks. Notably, SPS-CORT mice displayed significantly reduced freezing during the context test (Fig. 2F), along with a decrease in the percentage of total freezing duration (Fig. 2G), indicative of contextual amnesia. Finally, we assessed fear extinction across three consecutive days of context re-exposure. Control-Veh, Control-CORT, and SPS-Veh mice exhibited a significant reduction in freezing behavior by Day 3 relative to Day 1 (Fig. 2I), indicating successful extinction. In contrast, SPS-CORT mice failed to show this reduction, suggesting impaired fear extinction.

### Dorsal CA1 neurons from SPS-CORT group exhibit decreased input resistance, reduced action potential firing, and increased *I*_h_

**Fig. 3:**
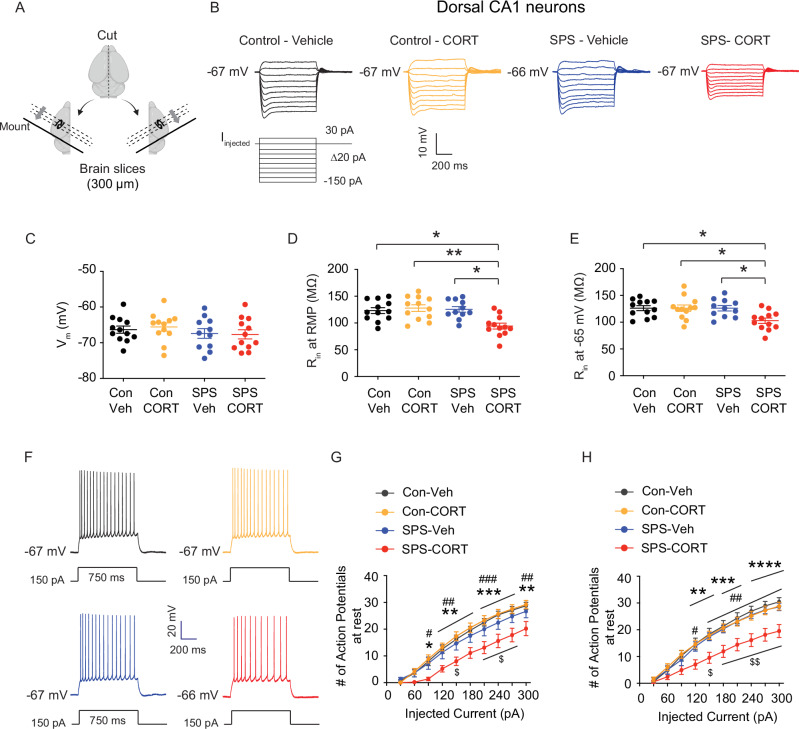
Dorsal CA1 neurons from SPS-CORT group exhibit decreased input resistance and reduced action potential firing. (**A**) Illustration of the preparation of dorsal hippocampal slices. (**B**) Representative voltage responses to a current step (-150 pA to 30 pA, Δ20 pA, 700 ms) at resting membrane potential. (**C**) Resting membrane potential. (**D**, **E**) Input resistance at RMP (**D**) and at -65 mV (**E**). The Kruskal-Wallis test was performed followed by Dunn’s multiple comparisons test. (**F**) Representative voltage responses to a depolarizing current step (150 pA; 750 ms) at RMP. (**G**, **H**) Number of action potentials at RMP (**G**) and at -65 mV (**H**). The Two-Way ANOVA followed by Tukey’s post-hoc test was performed. * indicates a comparison between the control-Veh and the SPS-CORT group. ^#^ indicates a comparison between the control-CORT and the SPS-CORT group. ^$^ indicates a comparison between the SPS-Veh and the SPS-CORT group. Data are expressed as mean ± SEM (cells/animal n values provided in Supplementary Table 1). *P < 0.05, **P < 0.01, ***P < 0.001, ****P < 0.0001, ^#^*P* < 0.05, ^##^*P* < 0.01, ^###^*P* < 0.001, ^$^*P* < 0.05, and ^$$^*P* < 0.01. Further statistical information is provided in the Supplementary Table 2. Panel A was created with BioRender.com.

**Fig. 4:**
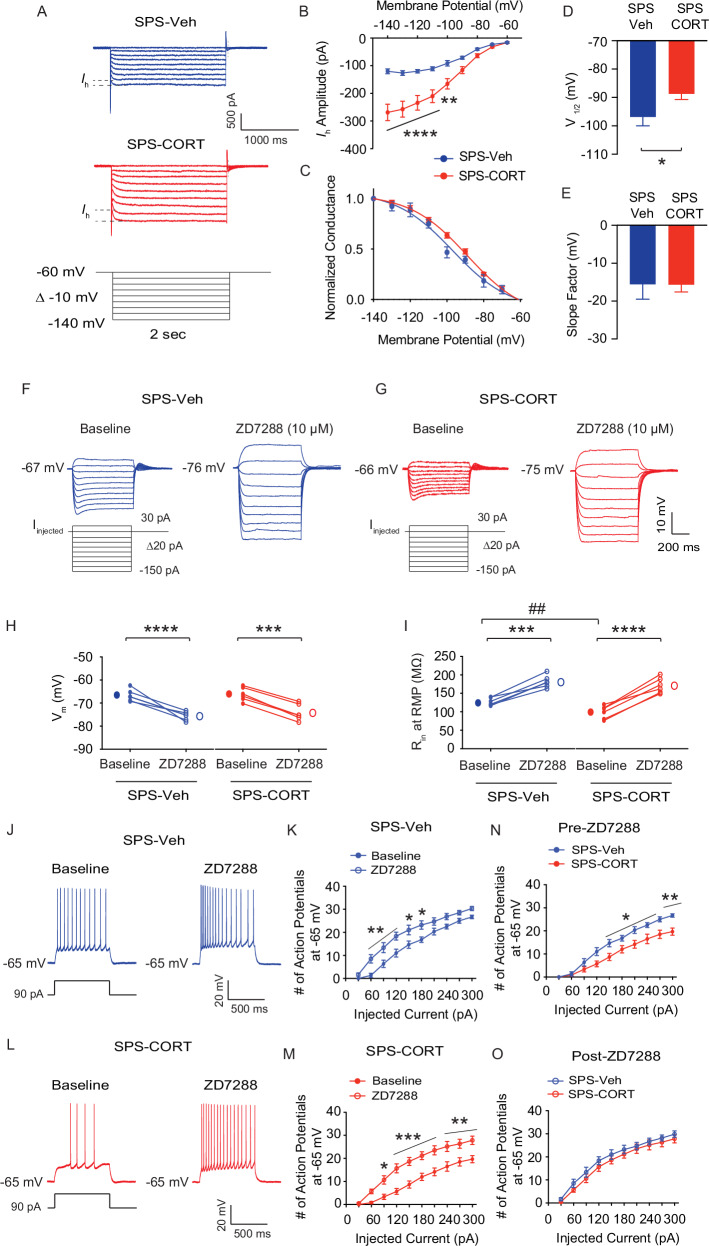
ZD7288 normalizes altered membrane properties in dorsal CA1 neurons of SPS-CORT mice. (**A**) Representative current responses to step voltage commands ranging from −140 mV to − 60 mV (Δ = -10 mV) at a holding potential of −60 mV in the dorsal CA1. The approximate position for determining the peak tail current is shown by gray vertical dashed lines. (**B**) *I*_h_ amplitude. The Two-Way ANOVA followed by Tukey’s post-hoc test was performed. (**C**) Voltage dependence of activation for *h* channel. The voltage dependence of activation (V_1/2_) for *h* channel was determined from tail currents (*I*_h_ / *I*_h_ max). The activation curve was fitted using a Boltzmann function, with the values of V_1/2_ and the slope factor. (**D**) V_1/2_ of *h* channel. The Mann-Whitney test was performed. (**E**) Slope factor. (**F**, **G**) Representative voltage responses to a current step (-150 pA to 30 pA, Δ20 pA, 700 ms) at resting membrane potential in SPS-Veh (**F**) and SPS-CORT (**G**) groups. (**H**) Resting membrane potential before and after bath application of ZD7288 (10 μM). The Wilcoxon matched-pairs signed rank test was performed separately for the SPS-Veh and SPS-CORT groups (**I**) Input resistance at RMP before and after bath application of ZD7288. The Wilcoxon matched-pairs signed rank test was performed separately for the SPS-Veh and SPS-CORT groups. The Mann-Whitney test was performed between SPS-Veh (baseline) and SPS-CORT (baseline) groups. (**J, L**) Representative voltage responses to a depolarizing current step (90 pA; 750 ms) from a holding potential of -65 mV in SPS-Veh (**J**) and SPS-CORT (**L**) groups. (**K, M**) Number of action potentials at -65 mV before (baseline) and after ZD7288 treatment in SPS-Veh (**K**) and SPS-CORT (**M**). (**N, O**) Group comparison of action potential firing before (**N**) and after (**O**) ZD7288 treatment between SPS-Veh and SPS-CORT mice. The Two-Way ANOVA followed by Tukey’s post-hoc test was performed. * indicates comparison between baseline and ZD7288 (**H, I, K, M**). ^#^ indicates comparison between the SPS-Veh and SPS-CORT groups (**I, N**). Data are expressed as mean ± SEM (cells/animal n values provided in Supplementary Table 1). *P < 0.05, **P < 0.01, ***P < 0.001, ****P < 0.0001, and ^##^*P* < 0.01. Further statistical information is provided in the Supplementary Table 2.

Given that SPS-CORT mice exhibited impairments in short-term spatial working memory, contextual fear recall, and fear extinction, we next investigated whether intrinsic membrane properties were altered in dorsal CA1 neurons. Using whole-cell current-clamp recordings, we assessed resting membrane potential (RMP), input resistance (R_in_), and action potential (AP) firing. Dorsal hippocampal slices were prepared following the 3-day extinction test (Fig. 3A). Compared to Control-Veh, Control-CORT, and SPS-Veh groups, SPS-CORT mice showed significantly reduced R_in_ at both RMP (Fig. 3D) and at a standardized membrane potential of −65 mV (Fig. 3E), while RMP itself did not differ across groups (Fig. 3C). Moreover, AP firing was markedly decreased in the dorsal CA1 neurons of SPS-CORT mice at both RMP (Fig. 3F, G) and −65 mV (Fig. 3F, H). These findings indicate a substantial reduction in the intrinsic excitability of dorsal CA1 neurons in SPS-CORT mice. Given previous reports of *I*_h_ enhancement following chronic stress paradigms such as chronic unpredictable stress [17] and chronic social defeat stress [15, 18], we next asked whether the reduced excitability observed in dorsal CA1 neurons of SPS-CORT mice might be associated with increased *I*_h_. To test this, we performed whole-cell voltage-clamp recordings to directly measure *I*_h_. Indeed, *I*_h_ was significantly elevated in dorsal CA1 neurons from SPS-CORT mice compared to SPS-Veh controls (Figs. 4A and 4B). Additionally, the voltage of half-maximal activation (V_₁/₂_) was shifted by approximately +8 mV, indicating that HCN channels in these neurons were more likely to open at depolarized membrane potentials (Fig. 4C, D). The slope factor, however, remained unchanged (Fig. 4C, E), suggesting no alteration in channel cooperativity or gating steepness. Given the significant increase in *I*_h_ observed in the SPS-CORT group, we next sought to confirm whether this enhancement was mediated by HCN channels. To do so, we compared RMP, R_in_ at RMP, and FI at -65 mV before and after bath application of ZD7288 (10 µM), an HCN channel blocker, in SPS-Veh and SPS-CORT mice. ZD7288 significantly hyperpolarized the RMP in dorsal CA1 neurons from both groups, with no group difference observed post-treatment (Fig. 4F–H). Consistent with our earlier findings of reduced R_in_ at RMP in SPS-CORT neurons (Fig. 3D), baseline R_in_ was significantly lower in the SPS-CORT group (Fig. 4I). However, following ZD7288 application, R_in_ values converged between groups (Fig. 4F,G, I). Consistent with the increase in input resistance (Fig. 4I), bath application of ZD7288 significantly enhanced action potential firing in both SPS-Veh (Fig. 4J, K) and SPS-CORT groups (Fig. 4L, M). Prior to HCN channel blockade, dorsal CA1 neurons from SPS-CORT mice exhibited markedly reduced firing compared with SPS-Veh controls (Fig. 4N). However, following ZD7288 treatment, this difference was no longer significant (Fig. 4O). These findings indicate that the reduction in excitability observed in SPS-CORT mice is primarily mediated by enhanced HCN channel activity.

### HCN1 overexpression in SPS mice mimics behavioral impairments induced by post-CORT exposure

**Fig. 5:**
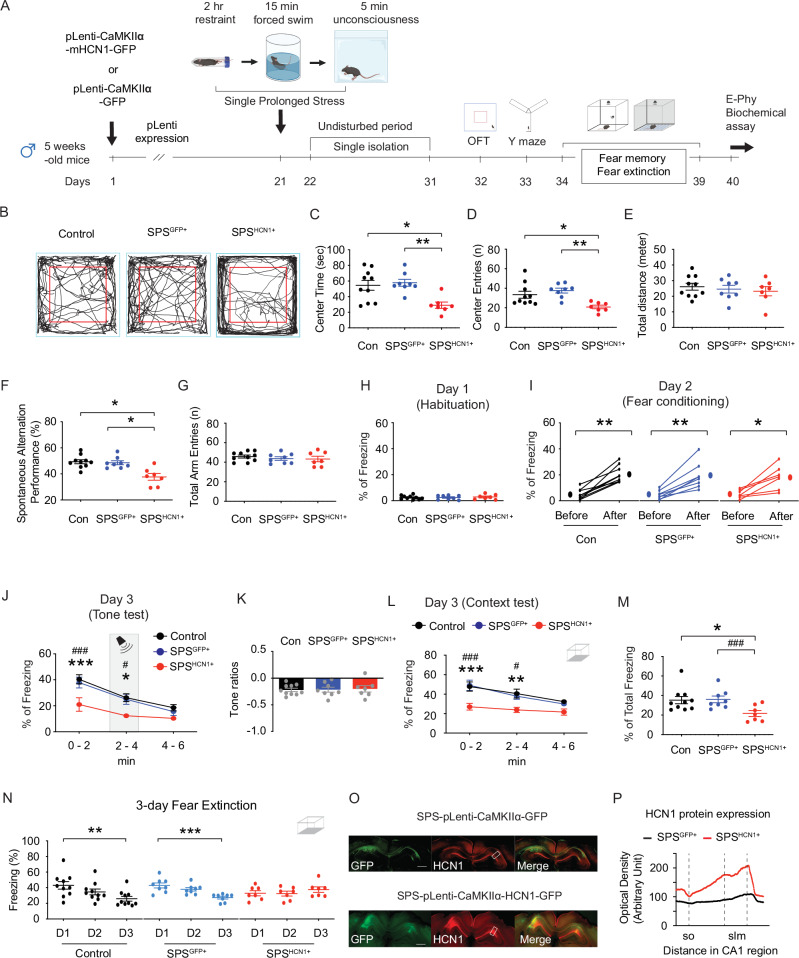
HCN1 overexpression in SPS mice induces PTSD-like behavioral deficits. (**A**) Schematic diagram of the experimental design. (**B**) Representative video tracking images of age-matched male mice during open field test. (**C**) Center time. (**D**) Center entries. The Kruskal-Wallis test was performed followed by Dunn’s multiple comparisons test. (**E**) Total traveled distance. (**F**) Percentage of spontaneous alternation performance among groups. (**G**) Total arm entries. (**H**) Habituation (**I**) Fear acquisition on Day 2. The Wilcoxon matched-pairs signed rank test was performed separately for the control, SPS^GFP+^, and SPS^HCN1+^ groups. (**J**) Percentage of freezing during the tone memory test. The Two-Way ANOVA followed by Tukey’s post-hoc test was performed. * indicates a comparison between the control and the SPS^HCN1+^ group. ^#^ indicates a comparison between the SPS^GFP+^ and the SPS^HCN1+^ group. (**K**) Tone ratios. (**L**) Percentage of freezing during the context memory test. The Two-Way ANOVA followed by Tukey’s post-hoc test was performed. * indicates a comparison between the control and the SPS^HCN1+^ group. ^#^ indicates a comparison between the SPS^GFP+^ and the SPS^HCN1+^ group. (**M**) The total percentage of freezing during a 6-minute context memory test. The Kruskal-Wallis test was performed followed by Dunn’s multiple comparisons test. (**N**) Fear extinction during the 3-day fear extinction test. The Kruskal-Wallis test was performed followed by Dunn’s multiple comparisons test separately for the control, SPS^GFP+^, and the SPS^HCN1+^ group. (**O**) Representative coronal sections of the dorsal hippocampus showing the areas of the CA1 region infected by lentivirus and immunolabeled with an antibody against HCN1. The rectangle box depicts the region of the slice used for quantification of optical density. Scale bar: 400 μm. (**P**) Quantification of HCN1 protein expression. Data are expressed as mean ± SEM (animal n values provided in Supplementary Table 1). *P < 0.05, **P < 0.01, ***P < 0.001, ^#^*P* < 0.05, and ^###^*P* < 0.001. Further statistical information is provided in the Supplementary Table 2. Panel A was created with BioRender.com.

Because (1) SPS-Veh mice showed no behavioral deficits or alterations in intrinsic membrane properties, and (2) SPS-CORT mice exhibited impairments in spatial memory, fear recall, and fear extinction—alongside increased *I*_h_ and reduced neuronal excitability—we next asked whether overexpression of HCN1 alone could reproduce the behavioral phenotype observed in SPS-CORT mice. To address this, five-week-old male mice received lentiviral injections expressing either GFP (control) or HCN1 under the CaMKIIα promoter. After allowing three weeks for viral expression, mice were subjected to the SPS procedure. Following a 10-day undisturbed period, mice underwent a battery of behavioral tests to evaluate anxiety-like behavior, exploratory activity, spatial working memory, and fear-related memory. In the open field test, mice with HCN1 overexpression following SPS (SPS^HCN1+^) exhibited a significant reduction in center time (Fig. 5B, C) and center entries (Fig. 5B, D), while total distance traveled did not differ across groups (Fig. 5B, E). In the Y-maze test, SPS^HCN1+^ mice exhibited a significantly lower spontaneous alternation performance (SAP) compared to both control and SPS^GFP+^ mice groups (Fig. 5F), while total arm entries remained unchanged (Fig. 5G), indicating impaired spatial working memory without alterations in locomotor activity. We next evaluated fear-associated memory using contextual fear conditioning. No group differences were observed during habituation on Day 1 (Fig. 5H) or during fear acquisition on Day 2 (Fig. 5I). In the tone re-exposure test, SPS^HCN1+^ mice showed reduced freezing behavior (Fig. 5J), although tone discrimination ratios were comparable across groups (Fig. 5K), suggesting that tone alone did not drive the freezing response. Strikingly, SPS^HCN1+^ mice displayed impaired contextual fear recall, as evidenced by reduced freezing during context re-exposure (Fig. 5L), along with a significant decrease in total freezing time (Fig. 5M). Furthermore, these mice failed to exhibit normal extinction of the conditioned fear response over three days of context re-exposure (Fig. 5N), indicating deficits in fear extinction learning. Following behavioral testing, viral targeting of the dorsal CA1 and HCN1 overexpression were confirmed by immunohistochemistry (Fig. 5O, P). Following preparation of dorsal hippocampal slices, we performed whole-cell recordings from dorsal CA1 neurons expressing the viral construct (Figure S2A). In current-clamp recordings, HCN1 overexpression resulted in membrane depolarization (Figure S2C), along with a significant reduction in R_in_ both at RMP (Figures S2B and S2D) and at −65 mV (Figures S2B and S2E). Action potential firing was also significantly reduced at both RMP (Figures S2F and S2G) and −65 mV (Figures S2F and S2H), confirming diminished neuronal excitability. In voltage-clamp recordings, we observed a marked increase in *I*_h_ in HCN1-overexpressing neurons (Figures S2I and S2J). Furthermore, the voltage of half-maximal activation (V₁/₂) was significantly depolarized (Figures S2K and S2L), while the slope factor remained unchanged (Figures S2K and S2M), indicating altered activation threshold without changes in gating kinetics.

### HCN1 deletion in dCA1 prevents SPS-CORT–induced behavioral and neuronal deficits

**Fig. 6:**
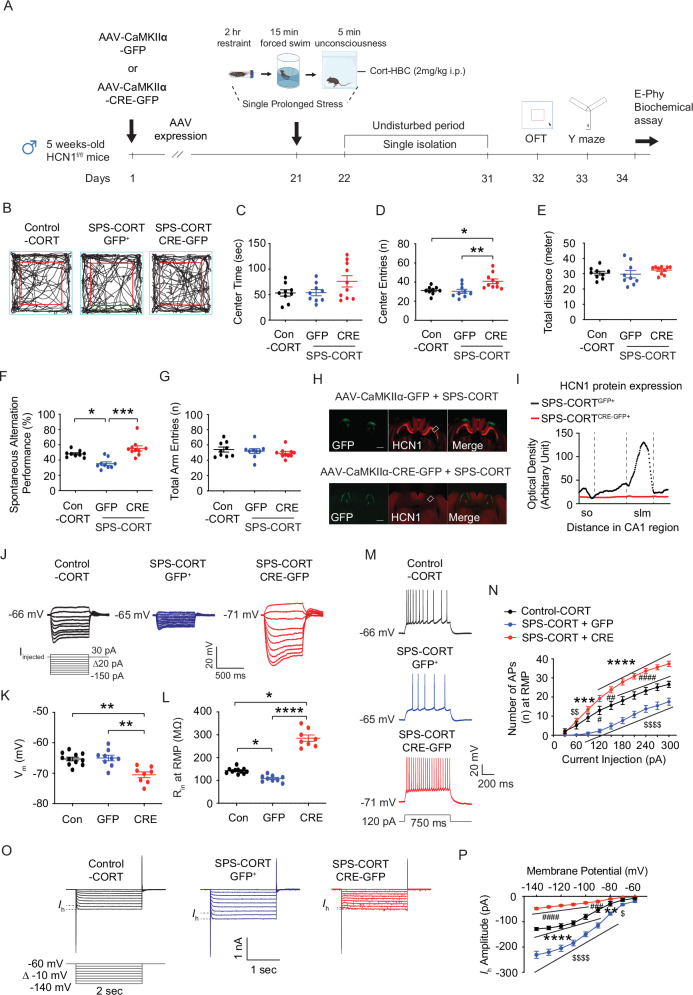
HCN1 deletion in dorsal CA1 neurons of SPS-CORT mice normalizes SPS-CORT-induced spatial working memory. (**A**) Schematic diagram of the experimental design. (**B**) Representative video tracking images of age-matched male mice during open field test. (**C**) Center time. (**D**) Center entries. The Kruskal-Wallis test was performed followed by Dunn’s multiple comparisons test. (**E**) Total traveled distance. (**F**) Percentage of spontaneous alternation performance among groups. The Kruskal-Wallis test was performed followed by Dunn’s multiple comparisons test. (**G**) Total arm entries. (**H**) Representative coronal sections of the dorsal hippocampus showing the areas of the CA1 region infected by AAV and immunolabeled with an antibody against HCN1. The rectangle box depicts the region of the slice used for quantification of optical density. Scale bar: 400 μm. (**I**) Quantification of HCN1 protein expression. (**J**) Representative voltage responses to a current step (-150 pA to 30 pA, Δ20 pA, 700 ms) at resting membrane potential. (**K**) Resting membrane potential. The Kruskal-Wallis test was performed followed by Dunn’s multiple comparisons test. (**L**) Input resistance at RMP. The Kruskal-Wallis test was performed followed by Dunn’s multiple comparisons test. (**M**) Representative voltage responses to a depolarizing current step (120 pA; 750 ms) at RMP. (N) Number of action potentials at RMP. (**O**) Representative current responses to step voltage commands ranging from −140 mV to − 60 mV (Δ = -10 mV) at a holding potential of −60 mV in the dorsal CA1. (P) *I*_h_ amplitude. The Two-Way ANOVA followed by Tukey’s post-hoc test was performed. * indicates comparison between Con-CORT and SPS-CORT^GFP+^ groups. ^#^ indicates comparison between Con-CORT and SPS-CORT^Cre+^ group. ^$^ indicates comparison between SPS-CORT^GFP+^ and SPS-CORT^Cre+^ groups. Data are expressed as mean ± SEM (cells/animal n values provided in Supplementary Table 1). *P < 0.05, **P < 0.01, ***P < 0.001, ****P < 0.0001, ^#^*P* < 0.05, ^##^*P* < 0.01, ^###^*P* < 0.001, ^####^*P* < 0.0001, ^$^*P* < 0.05, ^$$^*P* < 0.01,and *P* < 0.0001. Further statistical information is provided in the Supplementary Table 2. Panel A was created with BioRender.com.

Because HCN1 overexpression in SPS mice reproduced the impairments (Fig. 5) seen in SPS-CORT mice (Figs. 1 and 2), we next examined whether HCN1 in the dorsal CA1 is necessary for these abnormalities. To address this, we injected AAV-CaMKII-GFP-Cre into the dCA1 of HCN1 floxed mice to delete the channel, while AAV-CaMKII-GFP served as a control. Following three weeks of viral expression, mice underwent SPS followed by CORT administration (CORT-HBC; 2 mg/kg, i.p.) and were then given a 10-day recovery period before behavioral testing (Fig. 6A) An additional control group (Con-CORT in HCN1 floxed mice) was included in this experiment. In the open field, SPS-CORT^Cre+^ mice showed no difference in center time compared to SPS-CORT^GFP+^ mice (Fig. 6C), but exhibited more center entries (Fig. 6D), while total distance traveled was unaffected (Fig. 6E). In the Y-maze, SPS-CORT^GFP+^ mice displayed reduced spontaneous alternation performance (Fig. 6F), consistent with impaired spatial working memory. In contrast, mice lacking HCN1 in dCA1 showed increased SAP (%) relative to SPS-CORT^GFP+^ mice and performed at levels comparable to CORT-treated control (Fig. 6F). The total number of arm entries did not vary across groups (Fig. 6G), confirming that locomotor activity was intact. Immunohistochemistry verified accurate viral targeting of dCA1 and effective HCN1 deletion in SPS-CORT^Cre+^ mice (Fig. 6H, I). To evaluate physiological consequences, we performed whole-cell recordings from dCA1 pyramidal neurons. SPS-CORT^GFP+^ neurons showed reduced input resistance compared with control-CORT neurons (Fig. 6J, L), while resting membrane potential remained unchanged (Fig. 6J, K). In contrast, SPS-CORT^Cre+^ neurons exhibited a hyperpolarized resting membrane potential (Fig. 6K) and increased input resistance (Fig. 6L). Consistent with these changes, SPS-CORT^GFP+^ neurons fired fewer action potentials, whereas neurons lacking HCN1 in SPS-CORT^Cre+^ mice displayed enhanced firing capacity (Fig. 6M, N). Voltage-clamp analysis further revealed that *I*_h_ was elevated in SPS-CORT^GFP+^ neurons, but this increase was abolished by HCN1 deletion (SPS-CORT^Cre+^).Together, these results demonstrate that HCN1 in dCA1 is not only sufficient to reproduce SPS-CORT-related behavioral and cellular abnormalities, but also necessary for their manifestation.

## Discussion

Our results show that stress exposure and glucocorticoid signaling can interact to disrupt hippocampal function in a way that affects memory. On their own, neither SPS nor corticosterone (CORT) altered the intrinsic properties of dorsal CA1 neurons or produced consistent behavioral changes. However, when combined, SPS and CORT led to a clear reduction in input resistance, lower firing output, and elevated *I*_h_. These physiological changes occurred alongside deficits in spatial working memory, contextual recall, and extinction learning—behaviors that mirror the cognitive dimensions of PTSD. Interestingly, these impairments emerged without a measurable increase in open-field anxiety, pointing to a selective vulnerability in memory-related processes rather than a broad enhancement of emotional reactivity. To test the mechanism, we manipulated HCN1 expression in the dorsal CA1 neurons. Overexpressing HCN1 in SPS mice reproduced the memory deficits observed in the SPS–CORT group and, in addition, induced anxiety-like behavior. This suggests that excessive HCN1 activity is sufficient to drive both cognitive and emotional dysfunction, depending on its magnitude or context. In contrast, conditional deletion of HCN1 prevented the memory impairments and normalized excitability, establishing that HCN1 is also necessary for these effects. The pharmacological data further support this conclusion: application of ZD7288 reversed the intrinsic abnormalities in SPS–CORT mice, confirming that aberrant HCN activity lies at the center of the observed pathology.

These results are consistent with the idea that stress primes hippocampal circuits, making them more sensitive to subsequent glucocorticoid exposure. This could help explain why SPS produces reliable outcomes in rats but less consistent results in mice. In rats, extinction deficits and contextual memory impairments after SPS are well established [5–7], whereas in mice the findings are often strain-specific or protocol-dependent [8–10]. Differences in HPA-axis dynamics or glucocorticoid receptor sensitivity may account for these discrepancies, although direct comparisons are scarce. Within this framework, we view SPS–CORT not as a replacement for the standard SPS model but as a refinement suited to mice—one that allows us to amplify glucocorticoid-driven mechanisms and test their contribution to hippocampal dysfunction.

A growing body of work implicates maladaptive glucocorticoid receptor (GR) signaling in stress-related cognitive dysfunction, particularly within the hippocampus, a region essential for contextual memory and regulation of the HPA axis [29–33]. Studies using the SPS model consistently show that trauma exposure alters GR expression, phosphorylation, and downstream signaling [22, 34, 35]. In fact, corticosterone given 7 days after SPS can rapidly upregulate GR target genes in the dorsal hippocampus, suggesting that stress leaves behind a hypersensitive state of GR signaling [36]. Additional evidence comes from a genetically selected rat line with blunted glucocorticoid responsiveness, which displays multiple PTSD-like traits, including impaired extinction, smaller hippocampal volume, and disrupted REM sleep [37]. Building on this framework, we previously found that acute corticosterone treatment significantly increases HCN1 protein expression and membrane-bound *I*_h,_ as measured by cell-attached recordings in dorsal CA1 pyramidal neurons [15]. This rapid effect is likely mediated by enhanced membrane trafficking via the auxiliary subunit tetratricopeptide repeat-containing Rab8b-interacting protein, as evidenced by increased membrane-bound *I*_h_ [15]. These data suggest that glucocorticoids not only exert transcriptional control over HCN1 but can also rapidly alter its membrane availability and function through post-translational mechanisms, providing a direct link between acute stress hormone exposure and immediate changes in hippocampal excitability. In our current model, SPS followed by corticosterone administration (SPS-CORT) produces a sustained increase in *I*_h_, leading to reduced intrinsic excitability of dorsal CA1 neurons. This hypoexcitability is associated with behavioral deficits in working memory and contextual fear extinction—phenotypes that are absent in SPS-only or corticosterone-only conditions. Our findings suggest that upregulation of *I*_h_ serves as a maladaptive form of intrinsic plasticity that functionally decouples dorsal CA1 from afferent inputs (entorhinal cortex, CA3), thereby impairing memory consolidation [18]. Our results go further by showing that targeted overexpression of *HCN1* in dorsal CA1 neurons of SPS mice alone reproduced the same behavioral impairments observed in SPS mice infused with corticosterone. This phenocopy strongly supports a model where excessive GR activation acts through HCN1 upregulation to cause hippocampal dysfunction [15]. This pathway is consistent with molecular evidence that corticosterone, acting through GR and PKA, enhances TRIP8b and HCN1 expression to suppress excitability in dorsal CA1 neurons [15], and it converges with independent findings that glucocorticoids rapidly inhibit CA1 activity via HCN channels [38].

A few limitations deserve mention. We restricted our experiments to juvenile male mice to stay consistent with our prior physiology studies [15, 24] and to reduce variability from estrous cycling. This choice, however, means we cannot address sex- or age-related differences, which should be examined in future work. In addition, while we identified HCN1 as a downstream effector of stress hormone exposure, we did not directly manipulate GR signaling, leaving the upstream steps to be clarified in later studies.

In conclusion, the SPS–CORT paradigm unmasks latent stress vulnerabilities in mice, leading to reproducible deficits in working memory and extinction that are tightly linked to HCN1-dependent changes in dorsal CA1 excitability. HCN1 overexpression reproduced these impairments, deletion rescued them, and pharmacological blockade normalized excitability. Although SPS–CORT does not capture the full behavioral spectrum of PTSD, it provides a tractable model for investigating how stress hormones alter hippocampal circuits and highlights HCN1 as a potential therapeutic target for alleviating memory-related symptoms in stress disorders.

## Supplementary information

Supplemental Material

## Data Availability

The data that support the findings of this study are available from the corresponding author upon reasonable request.
