# Supplementary material for: Effects of post-stress corticosterone on hippocampal excitability and behavior involving hyperpolarization-activated cation channel 1 function
Source: Transl Psychiatry. 2026 Feb 7;16:74. doi: 10.1038/s41398-026-03871-4 (PMC12916791; doi:10.1038/s41398-026-03871-4)
Supplement: Supplementary file 1 — Supplemental Material [file 41398_2026_3871_MOESM1_ESM.docx]

***Supplementary Information***

**Effects of post-stress corticosterone on hippocampal excitability and behavior involving hyperpolarization-activated cation channel 1 function**

**Materials and Methods**

**Drugs**

ZD7288 (Cat #1000) was obtained from TOCRIS. (2-Hydroxypropyl)-β-cyclodextrin (HBC; Cat# H107) and Corticosterone-HBC complex (Cat# C174) were obtained from Sigma-Aldrich.

**Viruses**

pLenti-CaMKIIα-GFP (#VB220317-1266jgr) and pLenti-CaMKIIα-HCN1-GFP (#VB220317-1258jak) were purchased from VectorBuilder (USA). AAV-CaMKIIα-GFP (#105541) and AAV-CaMKIIα-GFP-CRE (#105551) were purchased from Addgene. Viral titers and injection volumes are provided in the Supplementary Information (Table S3).

**Stereotaxic microinjection**

Stereotaxic microinjections were carried out following established protocols with minor modifications^18, 26^. Prior to surgery, animals received a subcutaneous injection of carprofen (5 mg/kg, Covetrus). Male C57BL/6J mice aged 5 weeks were anesthetized with 0.5 L/min oxygen and 2% isoflurane throughout the procedure. Using a Hamilton syringe with a 26s-gauge needle (Hamilton, USA), mice were bilaterally injected with either pLenti-CaMKIIα-GFP or pLenti-CaMKIIα-HCN1-GFP into the dorsal hippocampal CA1 region (-1.8 mm anterior-posterior, ±1.6 mm medial-lateral, -1.4 mm dorsal-ventral from the dura) for the overexpression of the *HCN1* gene. For the deletion of HCN1, mice were bilaterally injected with either AAV-CaMKIIα-GFP or AAV- CaMKIIα-GFP-CRE into the dCA1 region. The injected volume was 0.4 µl per hemisphere, delivered slowly over 5 minutes. Following infusion, the injection needle was maintained in place for at least an additional 5 minutes to allow for diffusion and prevent backflow. Following the stereotaxic injection, the mice were returned to their home cages, and their body weight was monitored regularly to assess recovery and well-being.

**Immunohistochemistry**

Immunohistochemistry was carried out as described previously^15, 18^. Viral-infected dorsal hippocampal slices (80 μm thick) were prepared using a freezing microtome and stored in a cryoprotectant solution containing 30 % sucrose, 30% ethylene glycol, 1% polyvinyl pyrrolidone, 0.05 M sodium phosphate buffer for immunohistochemistry. Sections were briefly rinsed in PBS buffer and incubated in 0.1% TritonX-100 for 30 min. Subsequently, slices were blocked in PBS solution containing 5% normal goat serum, 0.03% TritonX-100 for 1hr, and then incubated in primary antibody diluted in blocking solution overnight at 4^o^C. Slices were rinsed in PBS buffer and then incubated in secondary antibody for 1hr at room temperature. Primary antibody in this study was used as follow; rabbit-anti-HCN1 (1:500, Invitrogen, Cat # PA5-78675). Additional antibody details are provided in the Supplementary Information (Table S3).


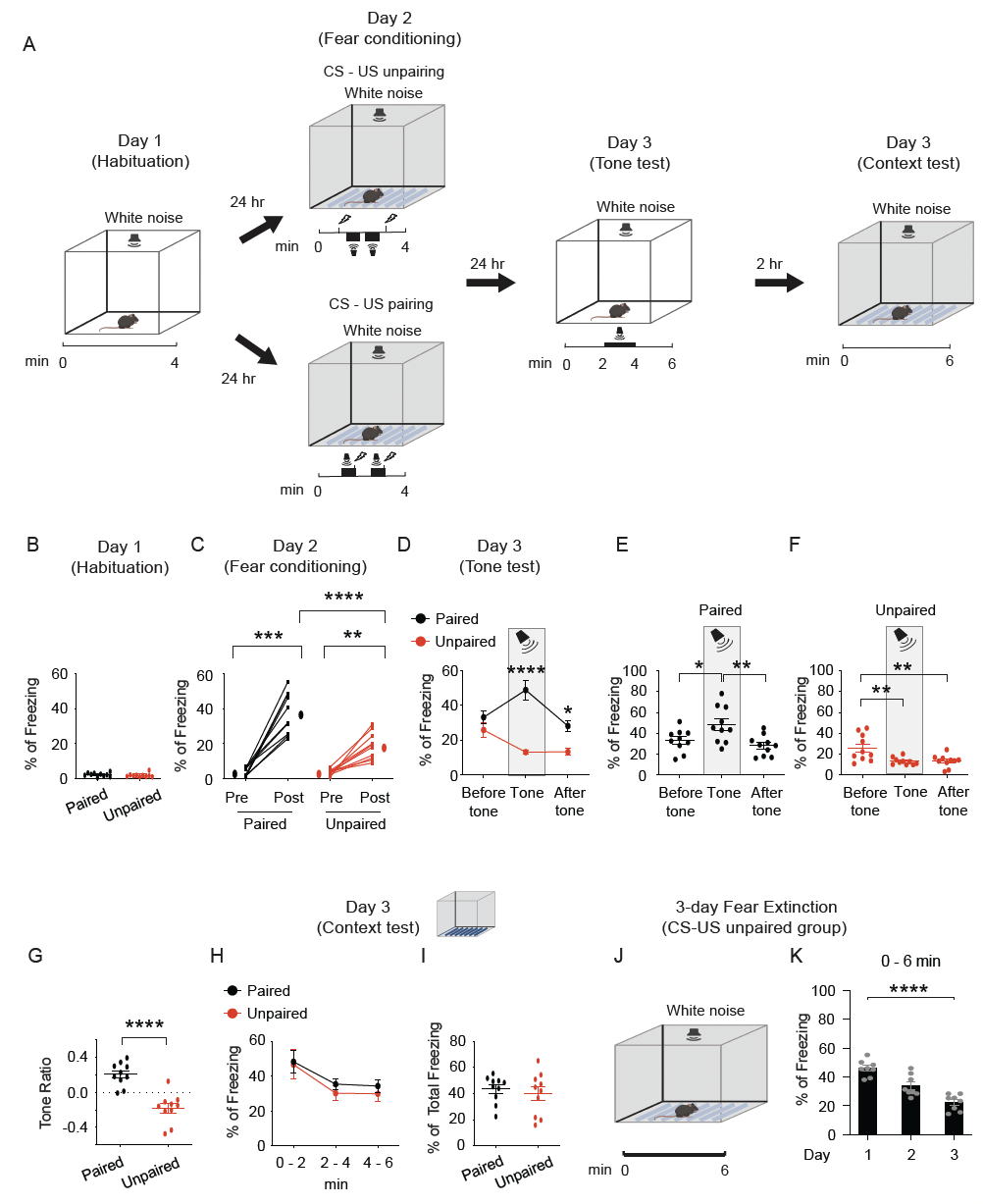


**Figure S1.Unpaired CS-US protocol reliably induces contextual fear learning and extinction.** (A) Illustration showing the contextual fear conditioning with paired and unpaired CS-US protocols in control mice. (B) Habituation session. (C) Fear acquisition in paired and unpaired CS-US groups. The Wilcoxon matched-pairs signed rank test was performed. (D) Percentage of freezing during the tone memory test. The tone was present during the 2-4-minute session of the experiment. (E) Tone served as a predictive factor in paired CS-US mice. (F) Tone did not elicit freezing in unpaired CS-US mice. (G) Tone ratios. (H) Percentage of freezing during the context memory test. (I) Total freezing during a 6-minute context memory test, showing comparable freezing between paired and unpaired CS-US mice. (J) Illustration of fear extinction experiment. (K) Fear extinction during the 3-day fear extinction test. The mouse was placed into the context for 6 minutes per day for 3 consecutive days. The Kruskal-Wallis test was performed followed by Dunn’s multiple comparisons test. Data are expressed as mean ± SEM. *P<0.05, **P<0.01, ***P<0.001, and ****P<0.0001. Panel A was created with BioRender.com.


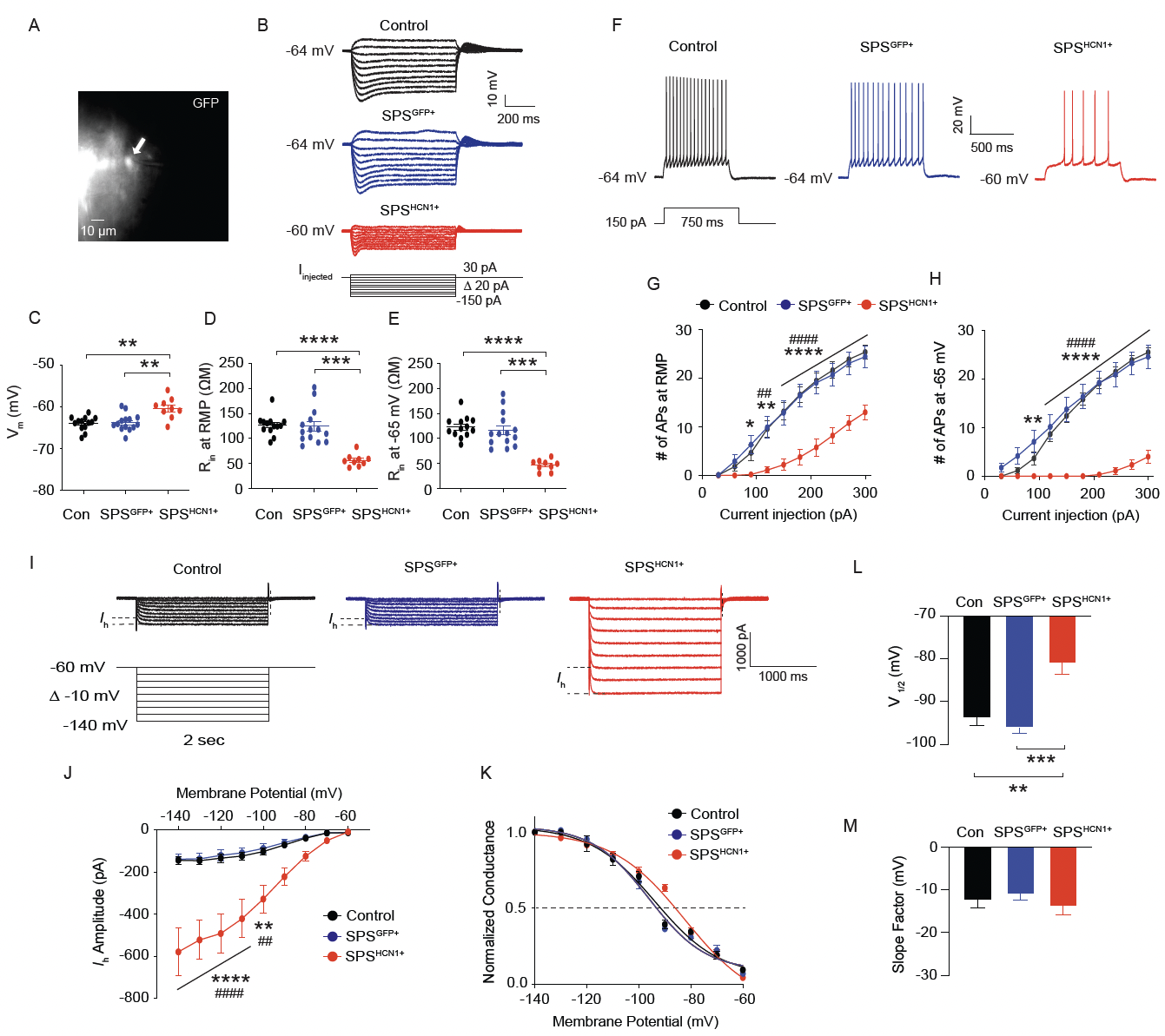


**Figure S2. Reduced neuronal excitability and enhanced *I*_h_ in dorsal CA1 neurons of SPS^HCN1+^ mice.**

(A) Photomicrograph of a representative recorded lentivirus-infected dorsal CA1 pyramidal neurons. The arrow indicates the recorded pyramidal neuron. (B) Representative voltage responses to a current step (-150 pA to 30 pA, Δ20 pA, 700 ms) at resting membrane potential. (C) Resting membrane potential. (D and E) Input resistance at RMP (D) and at -65 mV (E). (F) Representative voltage responses to a depolarizing current step (150 pA; 750 ms) at RMP. (G and H) Number of action potentials at RMP (G) and at -65 mV (H). (I) Representative current responses to step voltage commands ranging from −140 mV to − 60 mV (Δ = -10 mV) at a holding potential of −60 mV in the dorsal CA1. The approximate position for determining the peak tail current is shown by gray vertical dashed lines. (J) *I*_h_ amplitude. (K) Voltage dependence of activation for *h* channel. The voltage dependence of activation (V_1/2_) for *h* channel was determined from tail currents (*I*_h_ / *I*_h_ max). The activation curve was fitted using a Boltzmann function, with the values of V_1/2_ and the slope factor. (L) V_1/2_ of *h* channel. (M) Slope factor. The Kruskal-Wallis test was performed followed by Dunn's multiple comparisons test in (C), (D), (E), and (L). The Two-Way ANOVA followed by Tukey's post-hoc test was performed in (G), (H), and (J). * indicates a comparison between the control and the SPS^HCN1+^ group. ^#^ indicates a comparison between the SPS^GFP+^ and the SPS^HCN1+^ group. Data are expressed as mean ± SEM. *P<0.05, **P<0.01, ***P<0.001, ****P<0.0001, ^##^P<0.01, ^###^P<0.001, and ^####^P<0.0001.

Table S1. Descriptive statistics for all experimental groups.

| Figure | Group | Mean ±SEM | Cells (n) | Animals (n) |
| --- | --- | --- | --- | --- |
| Fig. 1C | Control-Veh | 41.68±4.81 | - | 9 |
| Center Time | Control-CORT | 36.45±3.68 | - | 9 |
|  | SPS-Veh | 45.48±5.34 | - | 9 |
|  | SPS-CORT | 43.81±4.82 | - | 9 |
| Fig. 1D | Control-Veh | 29.67±4.24 | - | 9 |
| Center Entries | Control-CORT | 26.44±2.66 | - | 9 |
|  | SPS-Veh | 27.11±2.34 | - | 9 |
|  | SPS-CORT | 29.78±3.74 | - | 9 |
| Fig. 1E | Control-Veh | 24.53±2.99 | - | 9 |
| Total distance | Control-CORT | 21.46±1.85 | - | 9 |
|  | SPS-Veh | 21.93±1.76 | - | 9 |
|  | SPS-CORT | 21.77±2.18 | - | 9 |
| Fig. 1G | Control-Veh | 53.60±2.08 | - | 9 |
| SAP (%) | Control-CORT | 48.22±2.67 | - | 9 |
|  | SPS-Veh | 44.44±2.41 | - | 9 |
|  | SPS-CORT | 36.84±1.90 | - | 9 |
| Fig. 1H | Control-Veh | 12.16±2.15 | - | 9 |
| SAR (%) | Control-CORT | 14.4±2.56 | - | 9 |
|  | SPS-Veh | 18.74±3.53 | - | 9 |
|  | SPS-CORT | 22.53±2.02 | - | 9 |
| Fig. 1I | Control-Veh | 44.44±4.91 | - | 9 |
| Total Arm Entries | Control-CORT | 41.78±4.44 | - | 9 |
|  | SPS-Veh | 41.89±3.90 | - | 9 |
|  | SPS-CORT | 41.44±4.60 | - | 9 |
|  |  |  |  |  |
| Fig. 2B | Control-Veh | 1.42±0.23 | - | 9 |
| Day1 (habituation) | Control-CORT | 2.02±0.46 | - | 9 |
|  | SPS-Veh | 1.72±0.27 | - | 9 |
|  | SPS-CORT | 2.36±0.43 | - | 9 |
| Fig. 2C | Control-Veh (Pre) | 2.32±0.56 | - | 9 |
| Day 2 ( Fear conditioning) | Control-Veh (Post) | 21.14±3.33 | - | 9 |
|  | Control-CORT (Pre) | 1.31±0.37 | - | 9 |
|  | Control-CORT (Post) | 24.10±4.77 | - | 9 |
|  | SPS-Veh (Pre) | 1.44±0.50 | - | 9 |
|  | SPS-Veh (Post) | 20.25±3.01 | - | 9 |
|  | SPS-CORT (Pre) | 2.54±0.66 | - | 9 |
|  | SPS-CORT (Post) | 19.11±2.95 | - | 9 |
| Fig. 2D | Control-Veh  (0-2) | 41.69±5.01 | - | 9 |
| Day 3 (Tone re-exposure test) | Control-Veh  (2-4) | 29.99±3.18 | - | 9 |
|  | Control-Veh  (4-6) | 28.49±4.35 | - | 9 |
|  | Control-CORT  (0-2) | 37.60±5.00 | - | 9 |
|  | Control-CORT  (2-4) | 27.80±4.25 | - | 9 |
|  | Control-CORT  (4-6) | 23.68±3.64 | - | 9 |
|  | SPS-Veh  (0-2) | 33.3±3.99 | - | 9 |
|  | SPSl-Veh  (2-4) | 26.35±3.00 | - | 9 |
|  | SPS-Veh  (4-6) | 21.14±3.23 | - | 9 |
|  | SPS-CORT  (0-2) | 48.83±8.65 | - | 9 |
|  | SPS-CORT  (2-4) | 26.77±4.03 | - | 9 |
|  | SPS-CORT  (4-6) | 21.56±3.03 | - | 9 |
| Fig. 2E | Control-Veh | -0.08±0.02 | - | 9 |
| Day 3 (Tone ratios) | Control-CORT | -0.05±0.03 | - | 9 |
|  | SPS-Veh | -0.01±0.05 | - | 9 |
|  | SPS-CORT | -0.08±0.08 | - | 9 |
| Fig. 2F | Control-Veh  (0-2) | 61.65±7.47 | - | 9 |
| Day 3 (Context re-exposure test) | Control-Veh  (2-4) | 48.24±5.10 | - | 9 |
|  | Control-Veh  (4-6) | 44.30±4.78 | - | 9 |
|  | Control-CORT  (0-2) | 60.34±8.35 | - | 9 |
|  | Control-CORT  (2-4) | 52.29±6.41 | - | 9 |
|  | Control-CORT  (4-6) | 50.78±6.46 | - | 9 |
|  | SPS-Veh  (0-2) | 57.42±4.54 | - | 9 |
|  | SPSl-Veh  (2-4) | 42.3±2.98 | - | 9 |
|  | SPS-Veh  (4-6) | 37.3±3.36 |  |  |
|  | SPS-CORT  (0-2) | 43.24±6.17 | - | 9 |
|  | SPS-CORT  (2-4) | 25.35±2.33 | - | 9 |
|  | SPS-CORT  (4-6) | 20.70±2.09 | - | 9 |
| Fig. 2G | Control-Veh | 52.71±5.59 | - | 9 |
| Day 3 (Total freezing) | Control-CORT | 54.97±6.40 | - | 9 |
|  | SPS-Veh | 47.76±2.68 | - | 9 |
|  | SPS-CORT | 30.64±2.97 | - | 9 |
| Fig. 2I | Control-Veh  (D1) | 42.69±4.37 | - | 9 |
| Fear extinction | Control-Veh  (D2) | 30.05±4.45 | - | 9 |
|  | Control-Veh  (D3) | 17.59±3.31 | - | 9 |
|  | Control-CORT  (D1) | 39.59±3.80 | - | 9 |
|  | Control-CORT  (D2) | 25.67±3.71 | - | 9 |
|  | Control-CORT  (D3) | 20.06±2.00 | - | 9 |
|  | SPS-Veh  (D1) | 39.09±3.08 | - | 9 |
|  | SPSl-Veh  (D2) | 33.14±2.64 | - | 9 |
|  | SPS-Veh  (D3) | 24.12±1.39 | - | 9 |
|  | SPS-CORT  (D1) | 32.74±2.14 | - | 9 |
|  | SPS-CORT  (D2) | 37.80±6.71 | - | 9 |
|  | SPS-CORT  (D3) | 36.27±5.03 | - | 9 |
|  |  |  |  |  |
| Fig. 3C | Control-Veh | -66.33±1.00 | 12 | 4 |
| Vm (mV) | Control-CORT | -65.60±1.17 | 12 | 4 |
|  | SPS-Veh | -67.41±1.35 | 11 | 4 |
|  | SPS-CORT | -67.65±1.27 | 12 | 4 |
| Fig. 3D | Control-Veh | 123.2±5.50 | 12 | 4 |
| Rin (MΩ) at RMP | Control-CORT | 127.8±6.18 | 12 | 4 |
|  | SPS-Veh | 125.5±5.24 | 11 | 4 |
|  | SPS-CORT | 94.22±5.54 | 12 | 4 |
| Fig.3E | Control-Veh | 126.1±4.61 | 12 | 4 |
| Rin (MΩ) at -65 mV | Control-CORT | 126.0±5.92 | 12 | 4 |
|  | SPS-Veh | 125.9±5.06 | 11 | 4 |
|  | SPS-CORT | 102.7±5.05 | 12 | 4 |
| Fig.3G | Control-Veh |  | 12 | 4 |
| FI at RMP | 30 pA | 0.75±0.51 |  |  |
|  | 60 pA | 3.50±1.35 |  |  |
|  | 90 pA | 8.00±1.62 |  |  |
|  | 120 pA | 12.58±1.60 |  |  |
|  | 150 pA | 16.25±1.55 |  |  |
|  | 180 pA | 19.58±1.51 |  |  |
|  | 210 pA | 22.58±1.41 |  |  |
|  | 240 pA | 25.33±1.42 |  |  |
|  | 270 pA | 27.08±1.36 |  |  |
|  | 300 pA | 28.75±1.34 |  |  |
|  | Control-CORT |  | 9 | 4 |
|  | 30 pA | 0.11±0.11 |  |  |
|  | 60 pA | 4.22±1.12 |  |  |
|  | 90 pA | 8.89±1.50 |  |  |
|  | 120 pA | 13.33±1.45 |  |  |
|  | 150 pA | 17.33±1.67 |  |  |
|  | 180 pA | 20.56±1.76 |  |  |
|  | 210 pA | 23.33±1.75 |  |  |
|  | 240 pA | 25.67±1.72 |  |  |
|  | 270 pA | 27.56±1.60 |  |  |
|  | 300 pA | 29.22±1.59 |  |  |
|  | SPS-Veh |  | 9 | 4 |
|  | 30 pA | 1.33±0.65 |  |  |
|  | 60 pA | 3.67±1.54 |  |  |
|  | 90 pA | 7.22±2.22 |  |  |
|  | 120 pA | 11.33±2.42 |  |  |
|  | 150 pA | 14.67±2.48 |  |  |
|  | 180 pA | 17.56±1.76 |  |  |
|  | 210 pA | 20.00±2.52 |  |  |
|  | 240 pA | 22.44±2.50 |  |  |
|  | 270 pA | 24.78±2.29 |  |  |
|  | 300 pA | 26.67±2.40 |  |  |
|  | SPS-CORT |  | 10 | 4 |
|  | 30 pA | 0 |  |  |
|  | 60 pA | 0.20±0.13 |  |  |
|  | 90 pA | 1.40±0.65 |  |  |
|  | 120 pA | 5.20±1.20 |  |  |
|  | 150 pA | 8.00±1.60 |  |  |
|  | 180 pA | 11.10±2.00 |  |  |
|  | 210 pA | 13.10±2.25 |  |  |
|  | 240 pA | 15.60±2.51 |  |  |
|  | 270 pA | 17.80±2.55 |  |  |
|  | 300 pA | 20.20±2.64 |  |  |
| Fig.3H | Control-Veh |  | 12 | 4 |
| FI at -65 mV | 30 pA | 0.58±0.29 |  |  |
|  | 60 pA | 4.83±1.38 |  |  |
|  | 90 pA | 10.25±1.95 |  |  |
|  | 120 pA | 14.67±2.22 |  |  |
|  | 150 pA | 18.33±2.17 |  |  |
|  | 180 pA | 21.33±2.10 |  |  |
|  | 210 pA | 24.33±2.00 |  |  |
|  | 240 pA | 26.92±1.88 |  |  |
|  | 270 pA | 28.92±1.66 |  |  |
|  | 300 pA | 30.42±1.57 |  |  |
|  | Control-CORT |  | 9 | 4 |
|  | 30 pA | 1.40±0.98 |  |  |
|  | 60 pA | 6.00±1.38 |  |  |
|  | 90 pA | 10.80±1.59 |  |  |
|  | 120 pA | 14.60±1.58 |  |  |
|  | 150 pA | 17.90±1.63 |  |  |
|  | 180 pA | 20.70±1.56 |  |  |
|  | 210 pA | 23.00±1.75 |  |  |
|  | 240 pA | 25.20±1.71 |  |  |
|  | 270 pA | 27.20±1.73 |  |  |
|  | 300 pA | 28.80±1.55 |  |  |
|  | SPS-Veh |  | 9 | 4 |
|  | 30 pA | 1.25±0.79 |  |  |
|  | 60 pA | 5.00±1.62 |  |  |
|  | 90 pA | 8.75±2.01 |  |  |
|  | 120 pA | 13.75±1.66 |  |  |
|  | 150 pA | 17.38±1.42 |  |  |
|  | 180 pA | 20.25±1.31 |  |  |
|  | 210 pA | 23.13±1.30 |  |  |
|  | 240 pA | 25.50±1.21 |  |  |
|  | 270 pA | 27.38±1.28 |  |  |
|  | 300 pA | 28.50±1.33 |  |  |
|  | SPS-CORT |  | 10 | 4 |
|  | 30 pA | 0.50±0.50 |  |  |
|  | 60 pA | 2.40±1.05 |  |  |
|  | 90 pA | 4.90±1.64 |  |  |
|  | 120 pA | 7.00±1.77 |  |  |
|  | 150 pA | 9.50±2.07 |  |  |
|  | 180 pA | 11.90±2.26 |  |  |
|  | 210 pA | 14.40±2.30 |  |  |
|  | 240 pA | 16.10±2.41 |  |  |
|  | 270 pA | 18.10±2.40 |  |  |
|  | 300 pA | 19.50±2.46 |  |  |
|  |  |  |  |  |
| Fig. 4B | SPS-Veh |  | 8 | 4 |
| Ih amplitude (pA) | -60 mV | -16.29±3.06 |  |  |
|  | -70 mV | -22.95±2.24 |  |  |
|  | -80 mV | -40.26±4.06 |  |  |
|  | -90 mV | -71.26±5.27 |  |  |
|  | -100 mV | -91.13±10.08 |  |  |
|  | -110 mV | -110.89±7.55 |  |  |
|  | -120 mV | -119.98±6.50 |  |  |
|  | -130 mV | -126.23±8.75 |  |  |
|  | -140 mV | -120.14±8.69 |  |  |
|  | SPS-CORT |  | 8 | 4 |
|  | -60 mV | -16.22±2.67 |  |  |
|  | -70 mV | -31.76±3.82 |  |  |
|  | -80 mV | -63.68±7.22 |  |  |
|  | -90 mV | -115.78±12.60 |  |  |
|  | -100 mV | -165.49±18.73 |  |  |
|  | -110 mV | -210.18±22.89 |  |  |
|  | -120 mV | -234.11±26.50 |  |  |
|  | -130 mV | -257.24±28.80 |  |  |
|  | -140 mV | -268.92±29.84 |  |  |
| Fig. 4D | SPS-Veh | -96.88±3.13 | 8 | 4 |
| V_1/2_ (mV) | SPS-CORT | -88.75±1.94 |  |  |
| Fig. 4E | SPS-Veh | -15.58±3.93 | 8 | 4 |
| Slope factor(mV) | SPS-CORT | -15.67±1.91 |  |  |
| Fig. 4H | SPS-Veh |  | 6 | 4 |
| RMP | Baseline | -66.75±1.04 |  |  |
|  | ZD7288 | -75.60±0.75 |  |  |
|  | SPS-CORT |  | 6 | 4 |
|  | Baseline | -66.15±1.20 |  |  |
|  | ZD7288 | -74.14±1.43 |  |  |
| Fig. 4I | SPS-Veh |  | 6 | 4 |
| Rin at RMP | Baseline | 127.3±4.27 |  |  |
|  | ZD7288 | 181.80±6.68 |  |  |
|  | SPS-CORT |  | 6 | 4 |
|  | Baseline | 99.22±7.17 |  |  |
|  | ZD7288 | 171.0±8.42 |  |  |
| Fig.4K | SPS-Veh (baseline) |  | 6 | 4 |
| FI at -65 mV | 30 pA | 0 |  |  |
|  | 60 pA | 1.33±0.95 |  |  |
|  | 90 pA | 6.33±1.63 |  |  |
|  | 120 pA | 11.00±1.73 |  |  |
|  | 150 pA | 14.67±1.58 |  |  |
|  | 180 pA | 16.83±1.22 |  |  |
|  | 210 pA | 20.33±1.48 |  |  |
|  | 240 pA | 22.50±1.06 |  |  |
|  | 270 pA | 25.00±1.10 |  |  |
|  | 300 pA | 26.67±0.76 |  |  |
|  | SPS-Veh (ZD7288) |  |  |  |
|  | 30 pA | 1.67±0.67 |  |  |
|  | 60 pA | 8.50±1.80 |  |  |
|  | 90 pA | 13.33±2.17 |  |  |
|  | 120 pA | 18.33±1.75 |  |  |
|  | 150 pA | 21.00±2.08 |  |  |
|  | 180 pA | 23.17±1.85 |  |  |
|  | 210 pA | 24.67±1.63 |  |  |
|  | 240 pA | 26.83±1.35 |  |  |
|  | 270 pA | 28.50±0.99 |  |  |
|  | 300 pA | 30.33±0.92 |  |  |
| Fig.4M | SPS-CORT (baseline) |  | 6 | 4 |
| FI at -65 mV | 30 pA | 0 |  |  |
|  | 60 pA | 0.83±0.48 |  |  |
|  | 90 pA | 3.33±1.15 |  |  |
|  | 120 pA | 5.67±1.28 |  |  |
|  | 150 pA | 8.67±1.54 |  |  |
|  | 180 pA | 12.00±1.59 |  |  |
|  | 210 pA | 14.17±1.68 |  |  |
|  | 240 pA | 16.50±2.08 |  |  |
|  | 270 pA | 18.50±1.80 |  |  |
|  | 300 pA | 19.67±1.56 |  |  |
|  | SPS-CORT (ZD7288) |  |  |  |
|  | 30 pA | 0.50±0.34 |  |  |
|  | 60 pA | 5.67±1.02 |  |  |
|  | 90 pA | 10.67±1.84 |  |  |
|  | 120 pA | 15.67±1.94 |  |  |
|  | 150 pA | 18.67±1.75 |  |  |
|  | 180 pA | 21.17±1.70 |  |  |
|  | 210 pA | 23.50±2.01 |  |  |
|  | 240 pA | 25.17±2.17 |  |  |
|  | 270 pA | 26.33±1.94 |  |  |
|  | 300 pA | 27.83±1.83 |  |  |
| Fig.4N | SPS-Veh (baseline) |  | 6 | 4 |
| FI at -65 mV | 30 pA | 0 |  |  |
|  | 60 pA | 1.33±0.95 |  |  |
|  | 90 pA | 6.33±1.63 |  |  |
|  | 120 pA | 11.00±1.73 |  |  |
|  | 150 pA | 14.67±1.58 |  |  |
|  | 180 pA | 16.83±1.22 |  |  |
|  | 210 pA | 20.33±1.48 |  |  |
|  | 240 pA | 22.50±1.06 |  |  |
|  | 270 pA | 25.00±1.10 |  |  |
|  | 300 pA | 26.67±0.76 |  |  |
|  | SPS-CORT (baseline) |  | 6 | 4 |
|  | 30 pA | 0 |  |  |
|  | 60 pA | 0.83±0.48 |  |  |
|  | 90 pA | 3.33±1.15 |  |  |
|  | 120 pA | 5.67±1.28 |  |  |
|  | 150 pA | 8.67±1.54 |  |  |
|  | 180 pA | 12.00±1.59 |  |  |
|  | 210 pA | 14.17±1.68 |  |  |
|  | 240 pA | 16.50±2.08 |  |  |
|  | 270 pA | 18.50±1.80 |  |  |
|  | 300 pA | 19.67±1.56 |  |  |
| Fig.4O | SPS-Veh (ZD7288) |  | 6 | 4 |
| FI at -65 mV | 30 pA | 1.67±0.67 |  |  |
|  | 60 pA | 8.50±1.80 |  |  |
|  | 90 pA | 13.33±2.17 |  |  |
|  | 120 pA | 18.33±1.75 |  |  |
|  | 150 pA | 21.00±2.08 |  |  |
|  | 180 pA | 23.17±1.85 |  |  |
|  | 210 pA | 24.67±1.63 |  |  |
|  | 240 pA | 26.83±1.35 |  |  |
|  | 270 pA | 28.50±0.99 |  |  |
|  | 300 pA | 30.33±0.92 |  |  |
|  | SPS-CORT (ZD7288) |  | 6 | 4 |
|  | 30 pA | 0.50±0.34 |  |  |
|  | 60 pA | 5.67±1.02 |  |  |
|  | 90 pA | 10.67±1.84 |  |  |
|  | 120 pA | 15.67±1.94 |  |  |
|  | 150 pA | 18.67±1.75 |  |  |
|  | 180 pA | 21.17±1.70 |  |  |
|  | 210 pA | 23.50±2.01 |  |  |
|  | 240 pA | 25.17±2.17 |  |  |
|  | 270 pA | 26.33±1.94 |  |  |
|  | 300 pA | 27.83±1.83 |  |  |
|  |  |  |  |  |
| Fig. 5C | Control | 54.51±6.33 | - | 10 |
| Center Time (sec) | SPS +GFP | 57.77±4.46 | - | 8 |
|  | SPS +HCN1 | 29.07±4.03 | - | 7 |
| Fig. 5D | Control | 33.40±3.58 | - | 10 |
| Center Entries (n) | SPS +GFP | 37.63±2.46 | - | 8 |
|  | SPS +HCN1 | 20.86±1.84 | - | 7 |
| Fig. 5E | Control | 26.11±2.19 | - | 10 |
| Total distance (meter) | SPS +GFP | 24.51±2.45 | - | 8 |
|  | SPS +HCN1 | 23.23±2.94 | - | 7 |
| Fig. 5F | Control | 49.35±1.52 | - | 10 |
| (SAP%) | SPS +GFP | 48.55±1.42 | - | 8 |
|  | SPS +HCN1 | 37.68±2.54 | - | 7 |
| Fig. 5G | Control | 45.80±1.52 | - | 10 |
| Total Arm Entries (n) | SPS +GFP | 43.75±1.79 | - | 8 |
|  | SPS +HCN1 | 43.29±2.78 | - | 7 |
| Fig. 5H | Control | 2.49±0.47 | - | 10 |
| Day 1 (Habituation) | SPS +GFP | 2.44±0.56 | - | 8 |
|  | SPS +HCN1 | 2.85±0.71 | - | 7 |
| Fig. 5I | Control (Pre) | 4.85±1.35 | - | 10 |
| Day 2 (Fear conditioning) | Control (Post) | 20.45±2.16 | - |  |
|  | SPS+GFP (Pre) | 4.59±1.16 | - | 8 |
|  | SPS+GFP (Post) | 19.08±3.46 | - |  |
|  | SPS-HCN1 (Pre) | 5.30±1.22 | - | 7 |
|  | SPS-HCN1 (Post) | 18.12±3.38 | - |  |
| Fig. 5J | Control  (0-2) | 40.36±3.51 | - | 10 |
| Day 3 (Tone re-exposure test) | Control  (2-4) | 26.22±3.03 | - |  |
|  | Control  (4-6) | 18.54±2.42 | - |  |
|  | SPS+GFP  (0-2) | 37.86±4.15 | - | 8 |
|  | SPS+GFP  (2-4) | 24.98±3.91 | - |  |
|  | SPS+GFP  (4-6) | 15.53±3.00 | - |  |
|  | SPS-HCN1  (0-2) | 20.97±5.22 | - | 7 |
|  | SPS-HCN1  (2-4) | 12.34±1.78 | - |  |
|  | SPS-HCN1  (4-6) | 10.31±1.27 | - |  |
| Fig. 5K | Control | -0.22±0.03 | - | 10 |
| Day 3 (Tone ratios) | SPS +GFP | -0.21±0.04 | - | 8 |
|  | SPS +HCN1 | -0.20±0.05 | - | 7 |
| Fig. 5L | Control  (0-2) | 47.78±4.77 | - | 10 |
| Day 3 (Context re-exposure test) | Control  (2-4) | 40.58±4.57 | - |  |
|  | Control  (4-6) | 32.28±2.28 | - |  |
|  | SPS+GFP  (0-2) | 49.11±5.29 | - | 8 |
|  | SPS+GFP  (2-4) | 37.88±3.22 | - |  |
|  | SPS+GFP  (4-6) | 29.89±2.23 | - |  |
|  | SPS-HCN1  (0-2) | 27.00±3.46 | - | 7 |
|  | SPS-HCN1  (2-4) | 23.85±2.61 | - |  |
|  | SPS-HCN1  (4-6) | 21.65±3.17 | - |  |
| Fig. 5M | Control | 35.33±3.77 | - | 10 |
| Day 3 (Total Freezing) | SPS +GFP | 36.04±3.42 | - | 8 |
|  | SPS +HCN1 | 21.66±3.11 | - | 7 |
| Fig. 5N | Control  (D1) | 43.01±4.78 | - | 10 |
| Fear extinction | Control  (D2) | 34.61±3.70 | - | 10 |
|  | Control  (D3) | 25.86±3.08 | - | 10 |
|  | SPS-GFP  (D1) | 42.91±3.21 | - | 8 |
|  | SPS-GFP  (D2) | 37.88±2.16 | - | 8 |
|  | SPS-GFP  (D3) | 27.27±1.76 | - | 8 |
|  | SPS-HCN1  (D1) | 32.92±3.26 | - | 7 |
|  | SPS-HCN1 (D2) | 32.42±3.10 | - | 7 |
|  | SPS-HCN1 (D3) | 37.57±3.50 | - | 7 |
|  |  |  |  |  |
| Fig. 6C | Control-CORT | 53.24±6.13 | - | 9 |
| Center Time (sec) | SPS-CORT- GFP | 53.75±6.08 | - | 9 |
|  | SPS-CORT-Cre | 76.12±11.09 | - | 10 |
| Fig. 6D | Control-CORT | 31.22±1.40 | - | 9 |
| Center Entries (n) | SPS-CORT- GFP | 30.33±2.16 | - | 9 |
|  | SPS-CORT-Cre | 40.80±2.53 | - | 10 |
| Fig. 6E | Control-CORT | 30.33±1.23 | - | 9 |
| Total Distance (meter) | SPS-CORT- GFP | 29.69±2.43 | - | 9 |
|  | SPS-CORT-Cre | 32.34±0.82 | - | 10 |
| Fig. 6F | Control-CORT | 48.20±1.43 | - | 9 |
| (SAP%) | SPS-CORT- GFP | 35.36±2.31 | - | 9 |
|  | SPS-CORT-Cre | 55.38±3.34 | - | 10 |
| Fig. 6G | Control-CORT | 54.11±3.23 | - | 9 |
| Total Arm Entries (n) | SPS-CORT- GFP | 52.00±3.22 | - | 9 |
|  | SPS-CORT-Cre | 49.20±1.97 | - | 10 |
| Fig. 6K | Control-CORT | -65.34±0.60 | 11 | 4 |
| Vm (mV) | SPS-CORT- GFP | -65.01±0.92 | 9 | 4 |
|  | SPS-CORT-Cre | -70.54±0.95 | 8 | 4 |
| Fig. 6L | Control-CORT | 142.4±3.69 | 11 | 4 |
| Rin (MΩ) at RMP | SPS-CORT- GFP | 108.3±4.74 | 9 | 4 |
|  | SPS-CORT-Cre | 285.3±13.82 | 8 | 4 |
| Fig.6N | Control-CORT |  | 10 | 4 |
| FI at RMP | 30 pA | 2.10±1.27 |  |  |
|  | 60 pA | 5.10±1.86 |  |  |
|  | 90 pA | 9.20±2.01 |  |  |
|  | 120 pA | 13.00±2.22 |  |  |
|  | 150 pA | 15.60±2.11 |  |  |
|  | 180 pA | 18.10±2.07 |  |  |
|  | 210 pA | 20.80±1.94 |  |  |
|  | 240 pA | 23.20±1.84 |  |  |
|  | 270 pA | 25.10±1.77 |  |  |
|  | 300 pA | 26.60±1.61 |  |  |
|  | SPS-CORT-GFP |  | 9 | 4 |
|  | 30 pA | 0 |  |  |
|  | 60 pA | 0.22±0.22 |  |  |
|  | 90 pA | 0.89±0.77 |  |  |
|  | 120 pA | 2.22±0.98 |  |  |
|  | 150 pA | 4.78±1.53 |  |  |
|  | 180 pA | 7.67±1.57 |  |  |
|  | 210 pA | 10.89±1.63 |  |  |
|  | 240 pA | 13.78±1.79 |  |  |
|  | 270 pA | 15.44±1.95 |  |  |
|  | 300 pA | 17.56±1.84 |  |  |
|  | SPS-CORT-Cre |  | 9 | 4 |
|  | 30 pA | 1.22±0.43 |  |  |
|  | 60 pA | 7.44±1.32 |  |  |
|  | 90 pA | 13.56±1.47 |  |  |
|  | 120 pA | 19.12±1.65 |  |  |
|  | 150 pA | 23.58±1.58 |  |  |
|  | 180 pA | 27.89±1.61 |  |  |
|  | 210 pA | 30.39±1.43 |  |  |
|  | 240 pA | 33.67±1.41 |  |  |
|  | 270 pA | 35.56±1.31 |  |  |
|  | 300 pA | 37.33±1.41 |  |  |
| Fig. 6P | Control-CORT |  | 8 | 4 |
| Ih amplitude (pA) | 60 mV | -7.65±2.25 |  |  |
|  | 70 mV | -14.08±5.30 |  |  |
|  | 80 mV | -28.79±9.18 |  |  |
|  | 90 mV | -53.48±10.12 |  |  |
|  | 100 mV | -80.42±10.02 |  |  |
|  | 110 mV | -104.81±8.27 |  |  |
|  | 120 mV | -117.11±8.92 |  |  |
|  | 130 mV | -124.50±7.01 |  |  |
|  | 140 mV | -128.59±5.57 |  |  |
|  | SPS-CORT-GFP |  | 4 | 2 |
|  | 60 mV | -18.79±4.75 |  |  |
|  | 70 mV | -32.04±3.88 |  |  |
|  | 80 mV | -67.79±6.13 |  |  |
|  | 90 mV | -114.63±9.83 |  |  |
|  | 100 mV | -149.75±9.96 |  |  |
|  | 110 mV | -184.10±11.41 |  |  |
|  | 120 mV | -204.61±12.21 |  |  |
|  | 130 mV | -220.69±12.27 |  |  |
|  | 140 mV | -230.32±14.07 |  |  |
|  | SPS-CORT-CRE |  | 8 | 4 |
|  | 60 mV | -3.08±1.26 |  |  |
|  | 70 mV | -3.54±1.32 |  |  |
|  | 80 mV | -10.12±3.10 |  |  |
|  | 90 mV | -19.89±3.62 |  |  |
|  | 100 mV | -25.46±4.16 |  |  |
|  | 110 mV | -31.90±3.95 |  |  |
|  | 120 mV | -36.03±3.64 |  |  |
|  | 130 mV | -41.50±3.73 |  |  |
|  | 140 mV | -47.84±4.39 |  |  |

Table S2. Summary of statistical analyses.

| **Figure** | **Statistical Tests** | **Comparison** | **H or F or W or U value** | **P value** |
| --- | --- | --- | --- | --- |
| Fig 1G | Kruskal-Wallis test | group | H (3,36)=16.45 | 0.0009 |
|  | Post-hoc | Con-Veh  vs.  SPS-CORT | n/a | 0.0004 |
| Fig 1H | Kruskal-Wallis test | group | H (3,36)=8.318 | 0.0399 |
|  | Post-hoc | Con-Veh  vs.  SPS-CORT | n/a | 0.0465 |
| Fig 2C | Wilcoxon matched-pairs signed rank test | Con-Veh  Before vs After | W=45 | 0.0039 |
|  | Wilcoxon matched-pairs signed rank test | Con-CORT  Before vs After | W=45 | 0.0039 |
|  | Wilcoxon matched-pairs signed rank test | SPS-Veh  Before vs After | W=45 | 0.0039 |
|  | Wilcoxon matched-pairs signed rank test | SPS-CORT  Before vs After | W=45 | 0.0039 |
| Fig 2F | Two-way ANOVA | Interaction | F (6,96)=0.2970 | 0.9370 |
|  |  | Time | F (2,96)=11.62 | <0.0001 |
|  |  | Group | F (3,96)=12.59 | <0.0001 |
| Fig 2G | Kruskal-Wallis test | group | H (3,36)=12.56 | 0.0057 |
|  | Post-hoc | Con-Veh  vs.  SPS-CORT | n/a | 0.0157 |
|  | Post-hoc | Con-CORT  vs.  SPS-CORT | n/a | 0.0108 |
| Fig 2I | Kruskal-Wallis test | Control-Veh | H (2,27)=13.14 | 0.0014 |
|  | Post-hoc | D1 vs. D2 | n/a | 0.1721 |
|  | Post-hoc | D1 vs. D3 | n/a | 0.0009 |
|  | Post-hoc | D2 vs. D3 | n/a | 0.2550 |
| Fig 2I | Kruskal-Wallis test | Control-CORT | H (2,27)=8.697 | 0.0129 |
|  | Post-hoc | D1 vs. D2 | n/a | 0.1257 |
|  | Post-hoc | D1 vs. D3 | n/a | 0.0125 |
|  | Post-hoc | D2 vs. D3 | n/a | >0.9999 |
| Fig 2I | Kruskal-Wallis test | SPS-Veh | H (2,27)=11.98 | 0.0025 |
|  | Post-hoc | D1 vs. D2 | n/a | 0.8551 |
|  | Post-hoc | D1 vs. D3 | n/a | 0.0021 |
|  | Post-hoc | D2 vs. D3 | n/a | 0.0616 |
| Fig 3D | Kruskal-Wallis test | Rin at RMP | H (3,47)=14.81 | 0.002 |
|  | Post-hoc | Con-Veh  vs.  SPS-CORT | n/a | 0.0183 |
|  | Post-hoc | Con-CORT  vs.  SPS-CORT | n/a | 0.0051 |
|  | Post-hoc | SPS-Veh  vs.  SPS-CORT | n/a | 0.0138 |
| Fig 3E | Kruskal-Wallis test | Rin at -65 mV | H (3,47)=11.52 | 0.0092 |
|  | Post-hoc | Con-Veh  vs.  SPS-CORT | n/a | 0.0294 |
|  | Post-hoc | Con-CORT  vs.  SPS-CORT | n/a | 0.0404 |
|  | Post-hoc | SPS-Veh  vs.  SPS-CORT | n/a | 0.0353 |
| Fig 3G | Two-way ANOVA | Interaction | F (27,360)=0.7333 | 0.8334 |
|  |  | pA | F (9,360)=108.4 | <0.0001 |
|  |  | Group | F (3,360)=42.28 | <0.0001 |
| Fig 3H | Two-way ANOVA | Interaction | F (27,360)=0.9616 | 0.5225 |
|  |  | pA | F (9,360)=101.2 | <0.0001 |
|  |  | Group | F (3,360)=42.53 | <0.0001 |
| Fig 4B | Two-way ANOVA | Interaction | F (8,126)=6.911 | <0.0001 |
|  |  | Vm | F (8,126)=45.88 | <0.0001 |
|  |  | Group | F (1,126)=105.3 | <0.0001 |
| Fig 4D | Unpaired t Test | V_1/2_:  SPS-Veh  vs.  SPS-CORT | t(13)=2.267  95% Confidential interval [0.388, 15.87]  R^2^=0.2833 | 0.0411 |
| Fig 4H | Wilcoxon matched-pairs signed rank test | SPS-Veh  Before vs After | W=-21 | 0.0312 |
|  | Wilcoxon matched-pairs signed rank test | SPS-CORT  Before vs After | W=-21 | 0.0312 |
| Fig 4I | Wilcoxon matched-pairs signed rank test | SPS-Veh  Before vs After | W=21 | 0.0312 |
|  | Wilcoxon matched-pairs signed rank test | SPS-CORT  Before vs After | W=21 | 0.0312 |
| Fig 4I | Mann-Whitney Test | SPS-Veh (Baseline)  Vs.  SPS-CORT (Baseline) | U=2  Difference:  Actual=-20.04  Difference:  Hodges-Lehmann=  -28.99 | 0.0087 |
| Fig 4K | Two-way ANOVA | Interaction | F (9,100)=0.9046 | 0.5244 |
|  |  | pA | F (9,100)=85.49 | <0.0001 |
|  |  | Group | F (1,100)=64.99 | <0.0001 |
| Fig 4M | Two-way ANOVA | Interaction | F (9,100)=1.667 | 0.1069 |
|  |  | pA | F (9,100)=52.80 | <0.0001 |
|  |  | Group | F (1,100)=112.6 | <0.0001 |
| Fig 4N | Two-way ANOVA | Interaction | F (9,100)=1.727 | 0.0926 |
|  |  | pA | F (9,100)=77.44 | <0.0001 |
|  |  | Group | F (1,100)=56.20 | <0.0001 |
| Fig 5C | Kruskal-Wallis test | Center Time | H (2,25)=10.52 | 0.0052 |
|  | Post-hoc | Con vs. SPS+GFP | n/a | >0.9999 |
|  | Post-hoc | Con vs. SPS+HCN1 | n/a | 0.0212 |
|  | Post-hoc | SPS+GFP  vs.  SPS+HCN1 | n/a | 0.0080 |
| Fig 5D | Kruskal-Wallis test | Center Entries | H (2,25)=7.704 | 0.0212 |
|  | Post-hoc | Con vs. SPS+GFP | n/a | >0.9999 |
|  | Post-hoc | Con vs. SPS+HCN1 | n/a | 0.0200 |
|  | Post-hoc | SPS+GFP  vs.  SPS+HCN1 | n/a | 0.1352 |
| Fig 5F | Kruskal-Wallis test | SAP (%) | H (2,25)=10.8 | 0.0045 |
|  | Post-hoc | Con vs. SPS+GFP | n/a | >0.9999 |
|  | Post-hoc | Con vs. SPS+HCN1 | n/a | 0.041 |
|  | Post-hoc | SPS+GFP  vs.  SPS+HCN1 | n/a | 0.0494 |
| Fig 5I | Wilcoxon matched-pairs signed rank test | Control  Before vs After | W=45 | 0.0039 |
|  | Wilcoxon matched-pairs signed rank test | SPS+GFP  Before vs After | W=36 | 0.0078 |
|  | Wilcoxon matched-pairs signed rank test | SPS+HCN1  Before vs After | W=28 | 0.0156 |
| Fig 5J | Two-way ANOVA | Interaction | F (4,66)=0.9028 | 0.4675 |
|  |  | Time | F (2,66)=22.60 | <0.0001 |
|  |  | Group | F (2,66)=13.74 | <0.0001 |
| Fig 5L | Two-way ANOVA | Interaction | F (4,66)=0.8116 | 0.5223 |
|  |  | Time | F (2,66)=8.979 | 0.0004 |
|  |  | Group | F (2,66)=14.97 | <0.0001 |
| Fig 5M | Kruskal-Wallis test | Total Freezing (%) | H (2,25)=8.200 | 0.0166 |
|  | Post-hoc | Con vs. SPS+GFP | n/a | >0.9999 |
|  | Post-hoc | Con vs. SPS+HCN1 | n/a | 0.0453 |
|  | Post-hoc | SPS+GFP  vs.  SPS+HCN1 | n/a | 0.0267 |
| Fig 5N | Kruskal-Wallis test | Control group | H (2,30)=8.632 | 0.0134 |
|  | Post-hoc | D1 vs. D2 | n/a | 0.5470 |
|  | Post-hoc | D1 vs. D3 | n/a | 0.010 |
|  | Post-hoc | D2 vs. D3 | n/a | 0.3285 |
| Fig 5N | Kruskal-Wallis test | SPS+GFP | H (2,24)=14.37 | 0.0008 |
|  | Post-hoc | D1 vs. D2 | n/a | >0.9999 |
|  | Post-hoc | D1 vs. D3 | n/a | 0.0011 |
|  | Post-hoc | D2 vs. D3 | n/a | 0.0106 |
| Fig 6D | Kruskal-Wallis test | Center Entries | H (2,28)=11.12 | 0.0038 |
|  | Post-hoc | Con-CORT vs.  SPS-CORT+GFP | n/a | >0.9999 |
|  | Post-hoc | Con-CORT vs.  SPS-CORT+CRE | n/a | 0.0227 |
|  | Post-hoc | SPS-CORT+GFP vs.  SPS-CORT+CRE | n/a | 0.0075 |
| Fig 6F | Kruskal-Wallis test | SAP % | H (2,38)=16.07 | 0.0003 |
|  | Post-hoc | Con-CORT vs.  SPS-CORT+GFP | n/a | 0.0351 |
|  | Post-hoc | Con-CORT vs.  SPS-CORT+CRE | n/a | 0.5012 |
|  | Post-hoc | SPS-CORT+GFP vs.  SPS-CORT+CRE | n/a | 0.0002 |
| Fig 6K | Kruskal-Wallis test | Vm | H (2,28)=13.41 | 0.0012 |
|  | Post-hoc | Con-CORT vs.  SPS-CORT+GFP | n/a | >0.9999 |
|  | Post-hoc | Con-CORT vs.  SPS-CORT+CRE | n/a | 0.0032 |
|  | Post-hoc | SPS-CORT+GFP vs.  SPS-CORT+CRE | n/a | 0.0044 |
| Fig 6L | Kruskal-Wallis test | Rin at RMP | H (2,28)=23.29 | <0.0001 |
|  | Post-hoc | Con-CORT vs.  SPS-CORT+GFP | n/a | 0.0283 |
|  | Post-hoc | Con-CORT vs.  SPS-CORT+CRE | n/a | 0.0339 |
|  | Post-hoc | SPS-CORT+GFP vs.  SPS-CORT+CRE | n/a | <0.0001 |
| Fig 6N | Two-way ANOVA | Interaction | F (18,250)=4.271 | <0.0001 |
|  |  | pA | F (9,250)=95.35 | <0.0001 |
|  |  | Group | F (2,250)=230.9 | <0.0001 |
| Fig 6P | Two-way ANOVA | Interaction | F (16,153)=19.25 | <0.0001 |
|  |  | Vm | F (8,153)=137.8 | <0.0001 |
|  |  | Group | F (2,153)=491.7 | <0.0001 |

Table S3. Viral vectors and antibodies used in this study.

| Reagent Type | Name | Source | Catalog ID | Concentration/  Amount |
| --- | --- | --- | --- | --- |
| Viral vector | pLenti-CaMKIIα-GFP | VectorBuilder | VB220317-1266jgr | Titer>10^9^ TU/ml,  0.4 µl per site |
| Viral vector | pLenti-CaMKIIα-HCN1-GFP | VectorBuilder | VB220317-1258jak | Titer>10^9^ TU/ml,  0.4 µl per site |
| Viral vector | AAV-CaMKIIα-GFP | Addgene | #105541 | Titer>10^13^ vg/mL,  0.4 µl per site |
| Viral vector | AAV-CaMKIIα-GFP-CRE | Addgene | #105551 | Titer>10^13^ vg/mL,  0.4 µl per site |
| Primary antibody | Anti-HCN1 (rabbit) | Invitrogen | PA5-78675 | 1:500 |
| Secondary antibody | Alexa Fluor 488 anti-rabbit | ThermoFisher Scientific | A-11008 | 1:1000 |
